# Nanomedicine beyond carriers — devices, cells & living therapeutics

**DOI:** 10.1007/s10544-026-00836-8

**Published:** 2026-09-08

**Authors:** Alessandro Grattoni, Marco Maria Paci, Noah Arnold, Santosh Aryal, Natalie Artzi, Gang Bao, Allan John R. Barcena, Einav Bentov-Arava, Elvin Blanco, Corrine Ying Xuan Chua, Bruna Corradetti, Alexander M. Cryer, Paolo Decuzzi, Marco De Giorgi, Cody Fell, Weiwei Gao, Balaji Govindaswamy, Wen Jiang, Goknur Kara, Michael R. King, Sunil Krishnan, William R. Lagor, Twan Lammers, Jessica Larsen, Francesco Manfredi, Devika Soundara Manickam, Katy Margulis, Marites Pasuelo Melancon, Silvie Meeuwissen, Michael A. Miller, Michael J. Mitchell, Samir Mitragotri, Anahita Mojiri, John P. Cooke, Rohan Palanki, Imonkanta Phukan, Xianwei Sun, Patrick S. Stayton, Eric A. Tanifum, Omid Veiseh, Sonia Villapol, Jingyi Wang, Ryan Williams, Ravit Yakobi Arancibia, Liangfang Zhang, Alexander Kabanov, Biana Godin

**Affiliations:** 1 Center for BioNanoengineering, Houston Methodist Hospital, Houston, TX USA; 2 Department of Chemical and Biomolecular Engineering, Clemson University, Clemson, SC USA; 3 Department of Pharmaceutical Sciences & Health Outcomes, University of Texas at Tyler, Tyler, TX USA; 4 Institute for Medical Engineering and Science, Massachusetts Institute of Technology, Cambridge, MA USA; 5 Department of Bioengineering, Rice University, Houston, TX USA; 6 Department of Interventional Radiology, The University of Texas MD Anderson Cancer Center, Houston, TX USA; 7 School of Pharmacy, Faculty of Medicine, Hebrew University of Jerusalem, Jerusalem, Israel; 8 Center for Precision Environmental Health, Baylor College of Medicine, Houston, TX USA; 9 Laboratory of Nanotechnology for Precision Medicine, Italian Institute of Technology, Genova, Italy; 10 Department of Integrative Physiology, Baylor College of Medicine, Houston, TX USA; 11 Aiiso Yufeng Li Family Department of Chemical and Nano Engineering, University of California San Diego, La Jolla, CA USA; 12 Department of Neurology, McGovern Medical School, UTHealth Houston, Houston, TX USA; 13 Department of Radiation Oncology, The University of Texas MD Anderson Cancer Center, Houston, TX USA; 14 Center for Neuroregeneration, Houston Methodist Research Institute, Houston, TX USA; 15 Department of Neurosurgery, Vivian L. Smith, UTHealth, Houston, TX USA; 16 Institute for Experimental Molecular Imaging and Nanomedicine, RWTH Aachen University Clinic, Aachen, Germany; 17 Ardena Oss BV, Nanomedicines, Oss, The Netherlands; 18 Department of Medicine, Division of Nephrology & Hypertension, Stony Brook University, Stony Brook, NY USA; 19 Department of Bioengineering, University of Pennsylvania, Philadelphia, PA USA; 20 John A. Paulson School of Engineering and Applied Sciences, Harvard University, Allston, MA USA; 21 Department of Cardiovascular Sciences, DeBakey Heart & Vascular Center, Houston Methodist Hospital, Houston, TX USA; 22 Department of Chemistry, The University of Texas at Austin, Austin, TX USA; 23 Department of Radiology, Texas Children’s Hospital and Baylor College of Medicine, Houston, TX USA; 24 Department of Bioengineering, University of Washington, Seattle, WA USA; 25 UNC Eshelman School of Pharmacy, University of North Carolina at Chapel Hill, Chapel Hill, NC USA

**Keywords:** Nanomedicine, Drug delivery, Immunotherapy, Gene and RNA therapeutics, Extracellular vesicles, Nanotheranostics

## Abstract

Nanomedicine has progressed far beyond its early role as an experimental drug-carrier toolbox and today stands as a clinically validated enabling technology. Liposomal formulations and albumin-bound nanoparticles have transformed cancer therapy, while lipid nanoparticle (LNP) platforms accelerated the rapid development and global deployment of SARS-CoV-2 mRNA vaccines—demonstrating how nanoscale engineering can reshape therapeutic response, manufacturing speed, and public health impact. With these successes as foundation, the field is rapidly expanding into more sophisticated delivery systems including cell-hitchhiking nanoparticles, biomimetic multivalent vaccines, implantable immunotherapy depots, high-loading polymeric micelles, organ-targeted gene and RNA carriers, and bio-hybrid extracellular vesicle and mitochondrial therapies. These platforms aim not only to transport drugs, but to modulate immunity, direct regeneration, and enable precision intervention at the cellular and molecular scale. This perspective summarizes the current landscape of biomedical nanotechnologies and outlines how their continued evolution positions nanomedicine as an enabling science driving the next generation of therapeutics.

## Introduction

Nanomedicine has matured from a conceptual approach for drug solubilization and passive tumor targeting into a foundational enabling technology with direct clinical impact. Over the past three decades, nanoscale engineering has contributed to FDA-approved cancer therapeutics, including liposomal doxorubicin (Barenholz 2012) and albumin-bound paclitaxel (Montero et al. 2011), and most notably, powered the unprecedented global deployment of lipid nanoparticle (LNP)–based mRNA vaccines against SARS-CoV-2 (Krammer 2020). These milestones demonstrate not only that nanomedicine works, but that it can fundamentally accelerate the translation of advanced therapeutics, shaping response profiles, reducing toxicity, and enabling molecular modalities previously infeasible in vivo. What began as a toolkit of carriers has evolved into a multidimensional scientific discipline capable of orchestrating immune activity, modulating gene expression, engineering cellular behavior, and creating hybrid bio-device interfaces for localized, durable therapy.

As the field advances, new platforms are emerging that blur the boundary between materials and biology. Cell-hitchhiking carriers and nanoparticles comprised of cell membrane elements exploit native trafficking pathways for controlled biodistribution. Multivalent vaccines and implantable cytokine-producing devices demonstrate that nanoscale systems can function as immunological actuators, not merely vehicles. Polymeric and mesoscale nanoparticles enable high-loading formulations and organ-specific targeting, while gene delivery platforms broaden nanomedicine into precise genome and transcript modulation. Nanotheranostics and molecular magnetic resonance imaging (MRI) contrast agents integrate imaging with treatment, enabling real-time visualization of drug distribution and response. Finally, extracellular vesicles and mitochondrial transfer therapeutics introduce bio-hybrid paradigms for metabolic rescue and neuroregeneration.

Conferences such as Nano Drug Delivery Systems (NanoDDS) 2025 serve as windows into this trajectory, highlighting leading edge developments across delivery science, immuno-engineering, polymer chemistry, organ-targeted therapeutics, nanotheranostics, and cell-derived therapeutic systems. The breadth of work presented at NanoDDS 2025 exemplifies how nanomedicine is evolving beyond drug carriers into an enabling science—one that interfaces deeply with immunotherapy, gene editing, regenerative medicine, oncology, infectious disease, and neurological repair.

**Fig. 1:**
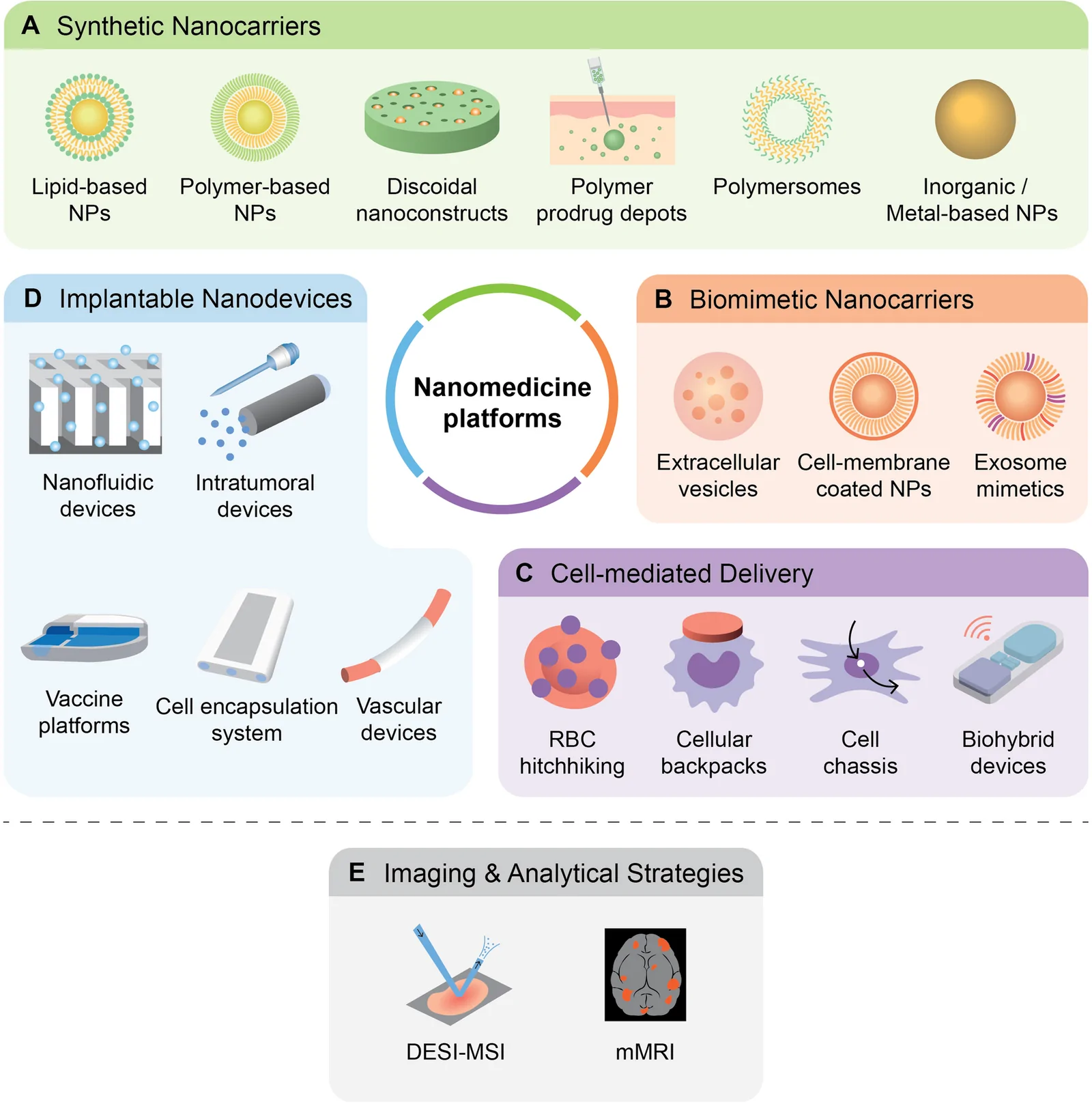
The expanded nanomedicine platform landscape. Nanomedicine has evolved from conventional nanoparticle-based drug delivery into a broader ecosystem of synthetic, biomimetic, cellular, implantable and analytical platforms. **A** | Synthetic nanocarriers, including lipid-based nanoparticles, polymer-based nanoparticles, discoidal nanoconstructs, polymer prodrug depots, polymersomes and inorganic or metal-based nanoparticles, enable controlled delivery of small molecules, proteins and nucleic acids. **B** | Biomimetic nanocarriers, such as extracellular vesicles, cell membrane–coated nanoparticles and exosome mimetics, exploit endogenous biological features to improve targeting, circulation and immune evasion. **C** | Cell-mediated delivery systems, including red blood cell hitchhiking, cellular backpacks, cell chassis and biohybrid devices, use living or cell-derived components to enhance biodistribution, localization and biological responsiveness. **D** | Implantable nanodevices, including nanofluidic devices, intratumoral devices, vaccine platforms, cell encapsulation systems and vascular devices, provide localized, sustained and programmable therapeutic administration. **E** | Imaging and analytical strategies, including desorption electrospray ionization mass spectrometry imaging and molecular magnetic resonance imaging, support spatial and functional characterization of nanomedicine biodistribution and biological responses. Together, these platforms illustrate the expansion of nanomedicine beyond passive carriers toward integrated, multifunctional and programmable therapeutic systems. NPs, nanoparticles; RBC, red blood cell; DESI-MSI, desorption electrospray ionization mass spectrometry imaging; mMRI, molecular magnetic resonance imaging

In this perspective, we outline the state-of-the-art in biomedical nanotechnologies across major thematic areas: (i) cellular and biomimetic delivery systems, (ii) nano-immunotherapeutics and tumor microenvironment engineering, (iii) polymeric and advanced material platforms, (iv) gene and RNA nanotherapies, (v) neurological nanomedicine and barrier-crossing strategies, (vi) nanotheranostics and imaging-guided delivery, (vii) extracellular vesicle- and organelle-based therapeutics, and (viii) manufacturing and quality-by-design frameworks for clinical translation. These advances illustrate a field transitioning from nanoscale tools to strategic, biology-integrated therapeutic platforms with increasing clinical relevance. This evolution reflects the emergence of a modular nanomedicine ecosystem integrating synthetic, biomimetic, cellular, implantable and living therapeutic systems (Fig. 1).

## Cellular carriers & hybrid bio-inspired delivery systems

### Cellular hitchhiking and backpacking strategies for drug delivery

Cellular hitchhiking and backpacking platforms leverage endogenous cell behaviors to enhance circulation, immune navigation, and tissue-specific delivery, transforming biological transport pathways into programmable tools for overcoming systemic barriers.

**Fig. 2:**
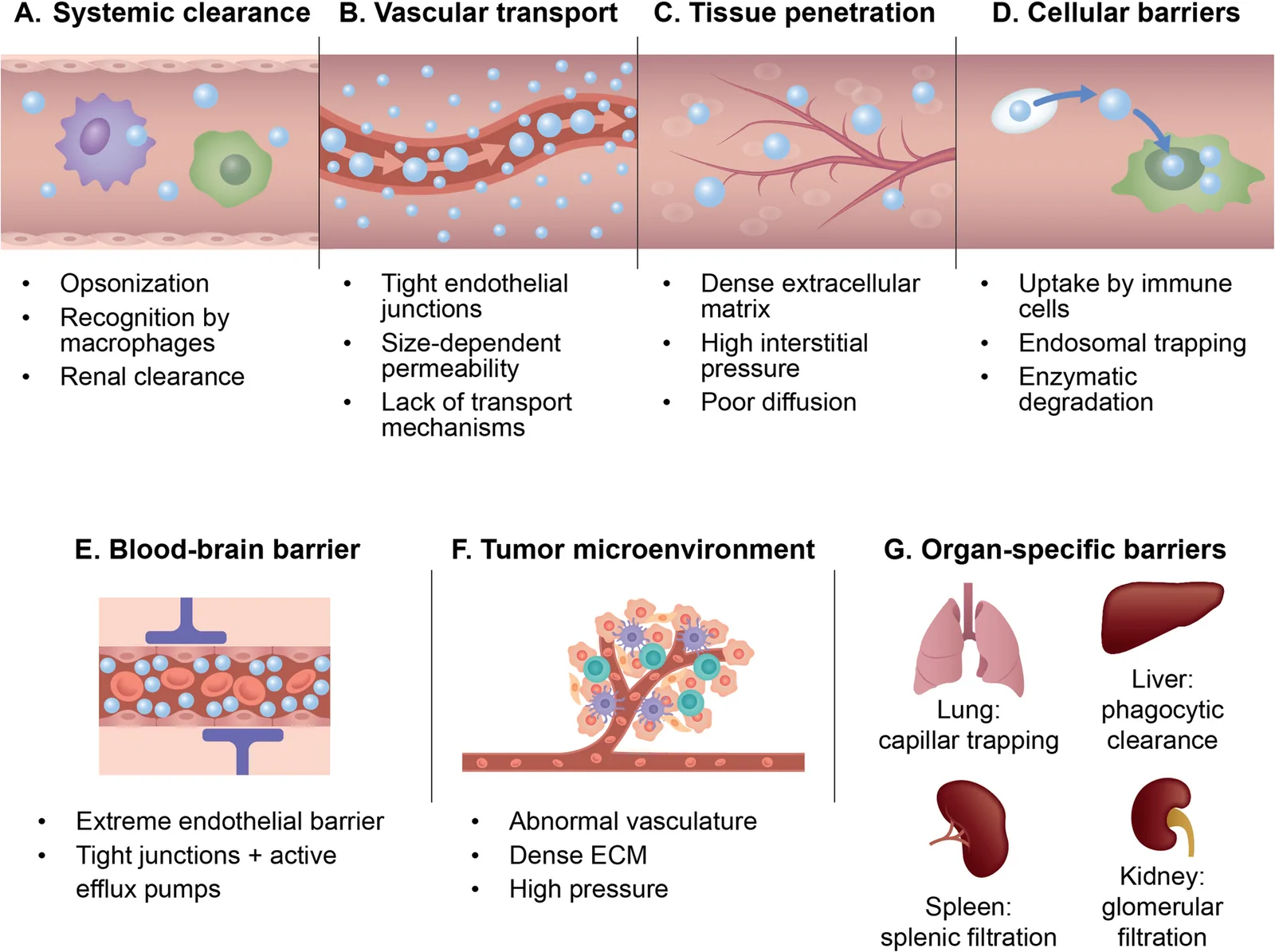
Biological transport barriers to nanomedicine. The systemic delivery of nanotherapeutics is limited by multiple biological barriers that restrict circulation, tissue access, cellular delivery and organ-specific biodistribution. **A** | Systemic clearance occurs through opsonization, recognition by macrophages and renal elimination of smaller constructs. **B** | Vascular transport is constrained by tight endothelial junctions, size-dependent permeability and limited active transport mechanisms, which regulate nanoparticle movement across the vessel wall. **C** | Tissue penetration is hindered by dense extracellular matrix, elevated interstitial pressure and poor diffusion through the tissue microenvironment. **D** | Cellular barriers include nonspecific uptake by immune cells, endosomal trapping and enzymatic degradation, all of which reduce intracellular therapeutic bioavailability. **E** | The blood–brain barrier represents an especially restrictive endothelial interface, characterized by tight junctions and active efflux pumps that limit access to the central nervous system. **F** | The tumor microenvironment combines abnormal vasculature, dense extracellular matrix and high interstitial pressure, producing heterogeneous and inefficient intratumoral distribution. **G** | Organ-specific barriers further shape biodistribution through lung capillary trapping, liver phagocytic clearance, splenic filtration and kidney glomerular filtration. These barriers underscore the need for nanomedicine strategies that are engineered for transport across multiple biological scales. ECM, extracellular matrix

Biological barriers of the human body often limit the transport of therapeutic nanoparticles (Mitchell et al. 2021). These barriers manifest in various forms ranging from the passive diffusion hurdle of epithelial tissues to active immune clearance of nanoparticles induced by the reticuloendothelial system (Tsoi et al. 2016) (Fig. 2). Various synthetic strategies are designed to overcome these barriers (Mitchell et al. 2021), however, their efficacy remains limited. In contrast to synthetic materials, endogenous cells possess intrinsic trafficking, deformation, and surveillance functions that allow them to traverse these same barriers with remarkable efficiency. For example, erythrocytes circulate for extended periods far beyond those exhibited by synthetic nanoparticles, and macrophages penetrate deeply into tumors, a goal that synthetic nanoparticles cannot readily achieve. These observations motivate the development of approaches that physically associate nanoparticles with circulating cells, either through surface adsorption (“hitchhiking”) or engineered attachment (“backpacking”), to improve delivery outcomes.

Nanoparticles hitchhiking on red blood cells (RBCs) exhibit reduced clearance in the liver and spleen and extended blood circulation. RBC-hitchhiking nanoparticles demonstrate substantial accumulation in the lungs due to contact-mediated dislodging of nanoparticles from the RBC surface. Formulations incorporating endothelial-permeabilizing agents further enhance penetration into distal lung regions, expanding the range of achievable pharmacological effects. The RBC-hitchhiking strategy is demonstrated for diverse cargoes including chemotherapy-loaded PLGA nanoparticles (Zhao et al. 2019), hydrogel nanoparticles (Park et al. 2025), and viral vectors (Brenner et al. 2018).

Cellular backpacks, discoidal polymer particles that integrate with immune cells, ride multiple cell types including macrophages (Shields et al. 2020), monocytes (Shields et al. 2020), T cells, neutrophils (Kumbhojkar et al. 2024) and B cells (Prakash et al. 2024). Backpacks regulate immune cell phenotype and activity, thus opening opportunities for treating multiple pathologies including cancer, multiple sclerosis (Kapate et al. 2023), and trauma (Kapate et al. 2024).

Combination of synthetic carriers and cells opens capabilities beyond those offered by either system alone. These hybrid systems offer the precision of synthetic designs, including controlled cargo loading and release, while preserving the navigation and targeting capabilities of the body’s own cells (Adebowale et al. 2024). Taken together, cellular hitchhiking and backpacking strategies illustrate a progression toward delivery systems that merge biological transport pathways with engineered nanoscale functionalities to achieve more selective, durable, and clinically adaptable therapeutic distribution.

Building upon these bio-integrated delivery approaches, membrane-coated and biomimetic nanovaccine platforms extend the same principles of biological interface engineering toward the activation and shaping of immune responses, as explored in Sect. 2.2.

### Multivalent nanovaccine platforms for broad immune protection

Membrane-coated and biomimetic nanovaccine platforms apply principles of natural antigen presentation to create multivalent, highly programmable immunomodulatory systems that address the limitations of conventional vaccines.

Coating natural cell membranes onto synthetic substrates has led to the development of cellular nanoparticles, CNPs, a nanomedicine platform with broad applications including multivalent nanovaccines (Fang et al. 2018). By presenting native epitopes from source cells while leveraging the tunable properties of synthetic cores, CNP nanovaccines can precisely modulate immune activation, offering a level of control and sophistication beyond conventional vaccine strategies. This combination of native membrane complexity with engineered nanoscale scaffolds enables highly configurable immunological engagement.

CNP nanovaccines present complex antigenic repertoires that are otherwise challenging to replicate. Cancer cell membrane-coated CNPs, for instance, recapitulate tumor antigen landscapes and elicit potent antitumor immunity (Fang et al. 2014; Krishnan et al. 2025; Kroll et al. 2017). Meanwhile, bacterial outer membrane vesicle, OMV, coated CNPs preserve pathogen-associated molecular patterns for immune presentation (Gao et al. 2015). Templated by the synthetic substrate, OMV-CNPs overcome limitations of traditional OMV vaccines such as heterogeneity, instability, and low adjuvant loading, yielding ultrasmall, uniform, and highly immunogenic nanostructures. As a result, they efficiently target lymph nodes and elicit robust, broad-spectrum immunity, including responses against antimicrobial-resistant strains (Bjånes et al. 2025; Gao et al. 2015).

CNP nanovaccines also evolve into nanotoxoids, where antigens are detained on nanoparticle surfaces and presented in native configurations without harmful effects, overcoming conflicting requirements of classical inactivated vaccines. For example, bacterial pore-forming toxins spontaneously incorporate into red blood cell membranes (Hu et al. 2013). In this design, the membrane sequesters toxins for safe immune presentation while maintaining their native conformation. Follow-up studies extend this concept using macrophage or neutrophil membranes to capture multiple virulence factors as immunogens, generating nanotoxoids that elicit robust protective immunity in mouse infection models (Wei et al. 2017; Zhou et al. 2022).

Looking ahead, CNP nanovaccines benefit substantially from advances in membrane genetic engineering. Precisely tailoring antigen profiles through engineered membranes enhances CNP specificity and potency (Jiang et al. 2020). The incorporation of orthogonal linkers into cell membranes further enables modular, plug-and-play conjugation strategies, transforming CNP nanotoxoids into rapidly customizable and scalable vaccine platforms with broad translational potential (Krishnan et al. 2024).

Beyond their immunological versatility, membrane-derived vaccines also point toward a broader trend: the engineering of cells themselves as therapeutic platforms. Section 2.3 explores this principle through implantable cell-based ‘living pharmacies’ that produce biologics on demand.

### Bioengineered cell-based nanotherapeutics and cytokine factories

Engineered cell-based nanotherapeutic systems extend the concept of bio-integrated delivery by using implanted, programmable cells as continuous sources of therapeutic molecules, creating sustained and highly localized treatment modalities that complement synthetic nanocarriers.

Cell-based living pharmacies represent an emerging paradigm in nanomedicine and drug delivery in which implanted cells, typically encapsulated within biomaterials, provide sustained and programmable production of biologic therapeutics. Recent clinical progress, including FDA approval of Neurotech’s ENCELTO, an encapsulated allogeneic ARPE-19 cell therapy implanted in the eye, anchors the feasibility of manufacturing, implanting, and regulating long-lived cell factories as medicines (ENCELTO 2025). These platforms deliver therapeutics locally at a disease site or secrete factors into circulation for systemic effect. In both cases, the approach shifts treatment from frequent injections to long-lived implants capable of maintaining steady-state exposure, reducing patient burden and improving adherence.

Most living pharmacy platforms integrate three engineering components. First, synthetic biology programs the cell chassis to control what is produced and when. Therapeutic cells produce antibodies, cytokines, or regenerative factors, and incorporate immunomodulatory strategies or safety features such as inducible control and kill switches to support containment and manage clinical risk (Hu et al. 2024; Imaichi-Kobayashi et al. 2023; Martin et al. 2020; Nash et al. 2022; Rivnay et al. 2025). Second, biomaterials and device interfaces shape the host response and regulate mass transport. Materials that reduce fibrotic encapsulation preserve long-term function but must still permit oxygen and nutrient exchange alongside therapeutic release (Bochenek et al. 2018; Lee et al. 2023; Vegas et al. 2016). Third, biohybrid device design adds retrievability and external control, including electro- or optogenetic actuation, which enables on-demand dose adjustment and rapid shutoff when needed (Bose et al. 2020; Huang et al. 2023; Rivnay et al. 2025).

Looking ahead, scaling and control represent key challenges. Volumetric efficiency must improve through higher cellular productivity and greater viable loading densities to support human-scale and multiplexed dosing within practical device footprints (Bose et al. 2020; Imaichi-Kobayashi et al. 2023; Lee et al. 2023; Vegas et al. 2016). In parallel, ensuring predictable dosing behavior, low-power operation, and durable performance in large-animal models will frame the translational path for these therapies (Hu et al. 2024; Huang et al. 2023; Krishnan et al. 2023; Rivnay et al. 2025; Vegas et al. 2016). Overcoming these challenges enables minimally invasive, dose-controllable, and durable platforms with the potential to transform therapeutic delivery across oncology, immunotherapy, and regenerative medicine.

Together, living pharmacy systems represent a natural bridge from cellular delivery toward immune-focused nanotechnologies, setting the stage for Sect. 3.1, which examines the structural design of nanomedicines engineered to modulate and activate immune responses.

## Nano-immunotherapeutics & tumor microenvironment engineering

### Structural nanomedicines for precision immunotherapy

**Fig. 3:**
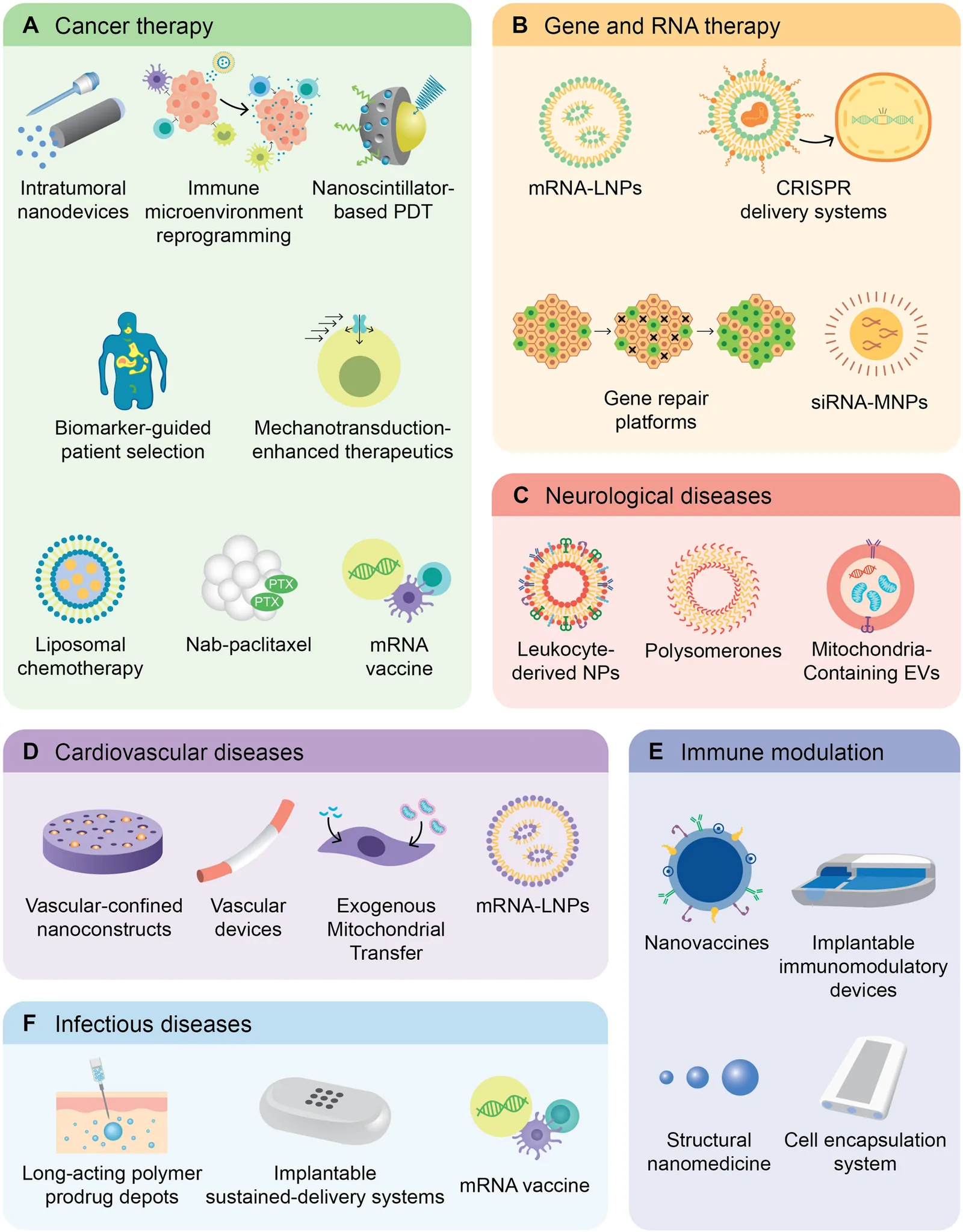
Therapeutic applications of nanomedicine. Nanomedicine platforms support a wide range of therapeutic applications by combining targeted delivery, controlled release, biological modulation and localized administration. **A** | Cancer therapy includes intratumoral nanodevices, tumor immune microenvironment reprogramming, nanoscintillator-based photodynamic therapy, biomarker-guided patient selection, mechanotransduction-enhanced therapeutics, liposomal chemotherapy, nab-paclitaxel and mRNA vaccines. **B** | Gene and RNA therapy use mRNA-loaded lipid nanoparticles, CRISPR delivery systems, gene repair platforms and siRNA nanoparticle formulations to modulate or correct disease-associated genetic programs. **C** | Neurological diseases may be addressed using leukocyte-derived nanoparticles, polymersomes and mitochondria-containing extracellular vesicles to improve delivery across restrictive neurovascular barriers and modulate neuroinflammation or neurodegeneration. **D** | Cardiovascular diseases include vascular-confined nanoconstructs, vascular devices, exogenous mitochondrial transfer and mRNA-LNP approaches designed to restore vascular, cellular or tissue function. **E** | Immune modulation includes nanovaccines, implantable immunomodulatory devices, structural nanomedicine and cell encapsulation systems that regulate immune responses through spatial and temporal control of therapeutic cues. **F** | Infectious diseases can be targeted using long-acting polymer prodrug depots, implantable sustained-delivery systems and mRNA vaccine platforms to improve durability, adherence and immune protection

Structural nanomedicines apply principles of precise molecular organization, spatial patterning, and material architecture to modulate immune activity with greater reproducibility, potency, and safety than conventional nanoparticle formulations. The convergence of these nanomedicine platforms has enabled a rapidly expanding spectrum of therapeutic applications across multiple disease areas (Fig. 3).

Structure dictates function, a principle central to chemistry, molecular biology, and protein engineering, yet historically underappreciated in nanomedicine. As nanomedicine expands into immunotherapy (Dosta et al. 2025; Irvine and Dane 2020), the need for structural control and reproducibility becomes increasingly prominent. Immunotherapies often require fine-tuned receptor engagement or coordinated activation of innate and adaptive pathways, where nanoscale arrangement critically influences efficacy. Accordingly, structural nanomedicine seeks to move beyond bulk characterization toward designs that explicitly encode spatial order, valency, and mechanical properties.

Current approaches that rely on population averages mask heterogeneity, making structure–function analysis difficult to interpret. By precisely controlling nanostructure architecture, researchers generate therapeutics with enhanced efficacy, safety, and on-target delivery, accompanied by reduced off-target effects (Mirkin et al. 2025a, b). In immunology, structural control over antigen and adjuvant density or spatial orientation strengthens cellular and humoral responses (Teplensky et al. 2023). For example, engineered ligand spacing enables selective engagement with antigen-presenting machinery or costimulatory receptors to enhance immunological potency (Sridhar et al. 2024). Similarly, materials incorporating cellular-responsive elements allow formulations to respond to extracellular or intracellular cues (Dosta et al. 2023; Zhang et al. 2020b), enabling selective drug release or multi-payload deployment based on disease stage or feedback signaling (Conde et al. 2015).

Structural definition also illuminates emergent biological processes. The paracrine transfer effect, whereby nanomaterials are internalized by one cell type then exported intact or as subunits to influence secondary recipient cells (Cryer et al. 2025; Paranandi et al. 2025), exemplifies how structure and biointerface interactions determine therapeutic outcomes. As analytical platforms expand alongside machine learning–driven design (Yamankurt et al. 2019), structural nanomedicine is positioned to generate immunotherapies with predictable, tunable, and mechanistically informed functions.

These structural design principles form a conceptual bridge to Sect. 3.2, where nanomaterials are engineered to reprogram macrophage behavior and reshape the tumor immune microenvironment with similar precision.

### Reprogramming macrophage behavior for cancer nanomedicine

Macrophage-targeted nanomedicine strategies redirect these highly plastic cells from clearance mediators into active therapeutic partners, enabling improved nanoparticle uptake, deeper tumor penetration, and enhanced antitumor immunity.

Macrophages represent a dual-edged axis in tumor biology, they can destroy malignant cells through phagocytosis (Zhou et al. 2021), yet also act as clearance sinks that remove systemically administered nanoparticles (Wang et al. 2024b). Reprogramming these cells within the tumor microenvironment enhances nanoparticle performance and modulates antitumor immunity with system-level impact. Tumor-associated macrophages, TAMs, frequently adopt immunosuppressive phenotypes that limit therapeutic success, and strategies that redirect their function shift macrophages from barriers into therapeutic enablers (Mantovani et al. 2022).

One approach, MUSIC, an ultrasound-guided platform, delivers the STING agonist cGAMP directly to antigen-presenting cells (Li et al. 2022). This spatially controlled activation amplifies type I interferon signaling, enhances dendritic cell priming, and reduces systemic cytokine toxicity that often limits STING-based therapeutics. MUSIC demonstrates that precise, localized innate activation overcomes limitations of systemic dosing.

Another innovation involves a microbial-inspired CD47–Listeriolysin O, LLO, construct that disrupts the “don’t eat me” axis to enable macrophage-mediated tumor cell phagocytosis (Schrank et al. 2025). CD47 blockade represents a key immune checkpoint beyond PD-1 and CTLA-4, and the fusion construct enhances phagocytosis by simultaneously engaging CD47 and supporting antigen processing pathways.

A third strategy, MARCO receptor blockade, reduces hepatic nanoparticle sequestration (Wang et al. 2024a). By limiting Kupffer cell uptake in the liver, MARCO inhibition increases nanoparticle bioavailability and improves tumor accumulation, resulting in enhanced therapeutic response. This approach illustrates how macrophage programming at the organ level can improve the pharmacokinetic profile of nanoparticle therapeutics.

Tumor macrophages can also be used as anchors for retaining therapeutics in the lesions with low vascularization patterns as discussed in 3.3.

### Targeting macrophages as a therapeutic strategy in hypovascularized tumor lesions

Macrophages can also be exploited to retain nanomedicines in the hypovascularized tumor lesions for enhanced therapeutic efficacy. As an example, breast cancer liver metastasis (BCLM) remains a major clinical challenge despite advances in breast cancer detection and treatment. The liver is a frequent site of metastasis, affecting 50–70% of patients with metastatic disease, and is associated with poor survival outcomes (median 4 months to 3 years). Current therapies, including surgery, systemic chemotherapy, immunotherapy, and targeted endocrine treatments, are often limited in effectiveness, particularly in patients with multiple metastatic lesions or aggressive subtypes such as triple-negative breast cancer (TNBC) (Ji et al. 2021; Xie and Xu 2019). A key barrier to effective treatment is the unique tumor microenvironment (TME) of BCLM. These lesions are typically hypovascularized and rely on existing liver vasculature rather than angiogenesis, resulting in poor perfusion and limited penetration of therapeutics. This vascularization pattern is uncommon compared to most solid tumors, and significantly limits the transport of molecules into the lesions. As a result, BCLM as other secondary liver tumors have poor permeation of molecules and are observed hypo-attenuating lesions (Daly et al. 1985; Tanei et al. 2016), which intravenously-injected contrast agents and macromolecules cannot permeate. This significantly contributes to physiological therapeutic resistance to both chemo- and immuno- therapies (Yan et al. 2023). Shifting the transport of a chemotherapeutic drug from the circulation towards macrophages in BCLM offers the potential to significantly improve therapeutic outcomes and survival benefits (Tanei et al. 2016). The outcomes are dependent on the macrophage phenotype, which can also be modulated (Leonard et al. 2017). These interactions can be examined through mathematical models of tumor–immune dynamics to help optimize therapeutic responses (Leonard et al. 2016, 2020). Integrating experimental data with computational modeling can therefore support realistic, predictive assessments of BCLM behavior during treatment, particularly by accounting for the availability of key cellular populations that retain and transport nanomedicine-associated high–molecular weight drugs within the tumor microenvironment.

Across the examples presented in 3.2 and 3.3, macrophages emerge as programmable regulators of nanomedicine biodistribution rather than passive obstacles. Future directions include combining macrophage reprogramming with nanoparticle vaccines, bispecific engagers, or localized immunomodulators to generate durable systemic tumor clearance.

Reprogramming macrophages systemically is one route to local immune control; another is to confine the immunomodulators themselves within the tumor, as Sect. 3.4 examines.

### Biodegradable intratumoral immunotherapy seed for localized multi-drug delivery

Intratumoral immunotherapy platforms that confine potent immunomodulators within the tumor microenvironment provide a powerful strategy to enhance efficacy while limiting systemic toxicity, particularly for multi-agent regimens that demand precise spatial and temporal control.

A central limitation in immuno-oncology is the narrow therapeutic window of synergistically potent combination regimens. Effective antitumor immunity often requires coordinated activation of innate sensing, co-stimulatory signaling, and immune checkpoint modulation, yet systemic administration frequently encounters dose-limiting toxicities (Dougan et al. 2021; Martins et al. 2019). Intratumoral immunotherapy seeks to overcome these constraints by maintaining high local drug concentrations while minimizing systemic exposure. Engineered delivery systems that localize immunomodulators within tumors have emerged as a robust framework to decouple biological efficacy from systemic off-target effects (Chua et al. 2020; Feng et al. 2025).

Intratumoral delivery has progressed from transient injections toward platforms that sustain drug release within solid tumors (Deeson et al. 2026; Liu et al. 2023b, 2024). By maintaining prolonged local exposure, these systems address heterogeneous drug distribution and rapid clearance into vasculature or lymphatics (Liu et al. 2021, 2023a). Engineered delivery architectures govern release kinetics through intrinsic material properties, including structural configuration, surface topography, polymer composition, and degradation dynamics, enabling temporal precision that direct injections alone cannot achieve (Fahey et al. 2026; Hood et al. 2016).

Future innovations increasingly focus on embedding bio-responsive elements that adapt release profiles to tumor-associated cues such as inflammation, enzymatic activity, or matrix remodeling. Such responsiveness is particularly relevant for immune pathways with narrow signaling thresholds, where premature or excessive exposure may trigger exhaustion or compensatory suppression. Drug-agnostic platforms that support sequential delivery of multi-pathway immunotherapies represent another critical direction. Early intratumoral release of innate activators, including STING and TLR7/8 agonists, may initiate inflammatory priming and antigen presentation, followed by phased delivery of co-stimulatory agonists, immune checkpoint inhibitors, or cytokines such as IL-12 to sustain effector responses and promote memory formation (Berraondo et al. 2018; Kota et al. 2024; Li et al. 2023).

Payload-agnostic architectures streamline clinical development by shifting dose-escalation considerations toward local pharmacokinetics and immune activation rather than systemic toxicities. In summation, localized platforms hold the potential to dismantle the traditional toxicity ceiling, transforming otherwise aggressive combination immunotherapies into safe, programmable, and clinically scalable interventions.

### Implantable nanomedicine platforms for local immune modulation and living therapeutics

Beyond injectable nanocarriers, implantable technologies incorporating engineered multifunctional nanoscale features have emerged as powerful platforms for timed and controlled delivery of therapeutic agents (Pons-Faudoa et al. 2023). These systems enable sustained, tunable, and compartmentalized modulation of complex biological environments (Chua et al. 2024). By integrating nanofluidics, biomaterials, and cell-based living therapeutics, implantable nanomedicine platforms can function as localized therapeutic microenvironments, supporting long-term regulation of immune responses, tissue regeneration, and cellular function.

In the context of nanofluidic modulation of the tumor immune microenvironment, one representative example is the nanofluidic drug-eluting seed (NDES), an implantable device designed for sustained and localized delivery of immunomodulatory agents directly within the tumor. By leveraging silicon–silicon carbide nanofluidic membranes with nanochannels with engineered electrostatics, NDES enables concentration-driven intratumoral release of immunotherapeutics and immune adjuvants over periods exceeding four weeks, while reducing systemic drug leakage and associated toxicities (Liu et al. 2021). In preclinical cancer models, intratumoral delivery of immune adjuvants or checkpoint inhibitors using these nanofluidic implants improved drug localization and persistence within the murine tumor models of triple negative breast cancer (TNBC) and pancreatic adenocarcinoma (KPC), promoted remodeling of the tumor immune microenvironment from immunologically “cold” to “hot,” enhanced therapeutic efficacy at one-quarter of the systemically effective dose, and abrogated systemic toxicity (Liu et al. 2023b, 2024). This localized strategy enables combinations of immunotherapeutics that are not feasible systemically because of dose-limiting toxicities. By generating potent local antitumor immunity, these platforms showed to induce systemic abscopal effects, support immunological memory, and contribute to the control of metastatic disease (Manfredi et al. 2026).

Building on the concept of localized immune microenvironment manipulation, subcutaneous bioengineered vaccine platforms have also emerged for the prevention and treatment of infectious, oncological, and autoimmune diseases. Among these, the NanoLymph platform extends nanofluidic technology toward implantable vaccine applications. Functioning analogously to an artificial lymph node, NanoLymph generates localized chemokine and chemoattractant gradients that selectively recruit immune cells into subcutaneous chambers vascularized by blood and lymphatic vessels (Viswanath et al. 2022). Following immune-cell recruitment, the platform enables in situ presentation of tolerogenic factors or immune activators together with defined antigens, thereby supporting prolonged antigen presentation and immune modulation (Kota et al. 2025). This approach can be applied to continuous vaccination against tumors through activation of tumor-draining lymph nodes, or alternatively to the induction of antigen-specific tolerance in autoimmune diseases such as type 1 diabetes. Continuous local immunostimulation through implantable nanofluidic platforms has been shown to enhance both prophylactic and therapeutic cancer vaccination responses (Kota et al. 2024).

Complementing these nanofluidic delivery technologies, the Neovascularized Implantable Cell Homing and Encapsulation (NICHE) device exemplifies a parallel strategy centered on cell-based therapeutic implantation combined with nanofluidic-controlled localized immunomodulation to prevent immune rejection of transplanted cells. The NICHE platform establishes a vascularized subcutaneous microenvironment capable of supporting engraftment of transplanted therapeutic cells while simultaneously providing in situ immunomodulation (Campa-Carranza et al. 2026) through transcutaneously replenishable controlled-release drug reservoirs. In rat and non-human primate preclinical models of type 1 diabetes, NICHE enabled long-term engraftment and function of transplanted allogeneic pancreatic islets while avoiding systemic immunosuppression (Campa-Carranza et al. 2025; Paez-Mayorga et al. 2020, 2022).

Collectively, these examples reflect a broader conceptual expansion in nanomedicine: from injectable drug carriers toward nanoengineered macroscopic technologies, including implants and scaffolds, capable of orchestrating complex biological responses and enabling temporal modification of therapeutic regimens. Current iterations that integrate synthetic biology circuits, bioelectronic control systems, and nanofluidic delivery architectures may ultimately enable biohybrid “living therapeutics” capable of sensing, computing, and responding to disease states in real time.

These principles of spatially and temporally defined immune modulation set the stage for Sect. 3.6, which examines radiation-triggered nanoscintillators designed to activate potent antitumor immunity within deep-seated tumors.

### X-ray-triggered nanoscintillator photodynamic therapy

Nanoscintillator-based photodynamic therapy leverages radiotherapy-activated nanoparticles to overcome depth limitations of conventional PDT, enabling controlled light production within deep-seated tumors to stimulate potent antitumor immunity.

Conventional photodynamic therapy, PDT, is restricted by shallow tissue penetration of visible and near-infrared light (Teraphongphom et al. 2017). As a result, many solid tumors, including pancreatic adenocarcinoma, remain inaccessible to clinically meaningful PDT doses. X-ray–activated nanoscintillators provide a strategy to bypass this limitation by converting penetrating radiation into localized intracellular light capable of activating photosensitizers (Sahin et al. 2025). And when coupled with immunotherapy, such externally activated nanoparticles can not only induce tumoricidal effects within the irradiated tumor but also stimulate a systemic immune response that causes regression of distant tumors that have not been irradiated (Sahin et al. 2020).

Nanoscintillator constructs such as pYSM (a core made of yttrium oxide doped with europium and a shell made of mesoporous silica containing methylene blue within and capped off with a polyethylene glycol epilayer) generate light upon X-ray irradiation and locally activate methylene blue photosensitizers to produce reactive oxygen species that induce tumor cell death (Sahin et al. 2025). Because light emission occurs within tumors, the system bypasses extrinsic optical delivery constraints and enables uniform PDT activation across tumor volumes that would otherwise be inaccessible. This approach integrates the spatial precision of radiotherapy with the immunogenic cell death profile characteristic of PDT, creating a hybrid modality often described as X-ray photodynamic therapy, XPDT. In syngeneic pancreatic cancer models, XPDT combined with anti-PD-1 therapy produces regression of both irradiated primary tumors and distant untreated lesions, demonstrating a robust abscopal immune response. The capacity to convert focal radiotherapy into systemic immune stimulation highlights XPDT as a unique platform for synergy between local cytotoxicity and checkpoint blockade. This profile is particularly compelling for pancreatic cancer, where immunologically cold microenvironments limit effectiveness of checkpoint inhibitors alone.

Future development will require optimization of nanoscintillator composition, radiation dose parameters, and photosensitizer loading to maximize quantum yield and enable clinical translation. Integrating XPDT into existing radiotherapy workflows may facilitate adoption, particularly for deep, hypovascular, or surgically inaccessible tumors where traditional PDT is not feasible.

Where XPDT shows that locally activated radiotherapy can drive systemic immune responses, the broader question of how to identify which patients are most likely to benefit from cancer nanomedicines — and how to track that benefit clinically — is taken up in Sect. 3.7.

### Toward clinical translation of cancer nanomedicine

Translational nanomedicine increasingly integrates precision frameworks that link patient-specific tumor biology with nanoparticle behavior, enabling more predictable treatment outcomes and the rational integration of nanotherapies into modern oncology.

Cancer nanomedicines continue to influence the clinical oncology landscape (Bhatia et al. 2022; Lammers 2024). Foundational formulations such as liposomal doxorubicin and albumin-bound paclitaxel improve tolerability and enhance therapeutic index, particularly when used in combination regimens. More recent platforms, including synergistic chemotherapy co-formulations and mRNA lipid nanoparticle vaccines, reinforce the capacity of nanotechnologies to deliver payloads with optimized ratios or encode neoantigen-targeted immune responses (Tzogani et al. 2020; Weber et al. 2024). These examples highlight the expanding functional diversity of nanomedicine within cancer treatment paradigms.

Promoting the performance and clinical impact of cancer nanomedicines requires strategic emphasis on patient stratification (Theek et al. 2014b; van der Meel et al. 2019). Lessons from targeted therapies demonstrate that biomarker-driven patient selection, based on receptor expression or mutational status, is critical for achieving meaningful response rates and successful progression into late-phase clinical trials (van der Meel et al. 2019). Analogously, tumor-targeted nanomedicines benefit from biomarkers that predict nanoparticle accumulation, immune contexture, or relevant features of the tumor microenvironment.

Tumor accumulation of therapeutic agents remains heterogeneous across patients and even within individual tumors. This variability extends not only to nanoparticle platforms but also to small molecules, antibodies, and cell-based therapies (Lammers 2024). As a result, tools that assess tumor access and drug delivery have broad relevance. Approaches range from direct theranostic imaging of nanoparticle accumulation using PET, CT, or MRI (Lee et al. 2017; Miedema et al. 2022), to indirect assessment via iron oxide nanoparticles, microbubble perfusion imaging, or contrast-enhanced ultrasound (Ramanathan et al. 2017; Theek et al. 2014a). Histopathological biomarkers, including tumor vascularization and stromal density, further aid in predicting whether nanomedicines can effectively accumulate within tumors (May et al. 2024).

Considering these factors, biomarkers for patient selection become instrumental for maximizing clinical success. Such tools help clinicians identify patients with a high likelihood of treatment benefit, guide investors in supporting predictable translational pathways, and ensure that clinical study populations possess characteristics aligned with therapeutic mechanism and delivery behavior.

Patient stratification, imaging, and microenvironmental profiling are translational tools; their utility ultimately depends on the carriers themselves. Section 4.1 turns to the polymeric chemistry that makes high-performance nanocarriers possible.

## Polymeric nanocarriers & advanced material systems

### Poly(2-oxazoline) micelles and polymer platforms for high-load drug delivery

Poly(2-oxazoline), POx, and poly(2-oxazine), POzi, polymers provide a chemically diverse and highly tunable class of materials that enable next-generation nanocarrier architectures, offering structural definition, formulation versatility, and performance advantages over traditional pharmaceutical excipients.

Poly(2-oxazolines), POx, and poly(2-oxazines), POzi, have rapidly advanced as versatile polymeric materials for pharmaceutics due to precise, scalable synthesis and exceptional monomer diversity (Lorson et al. 2018; Zahoranova and Luxenhofer 2021). These synthetic polyamides resemble polyvinylpyrrolidone, PVP, structurally and share features with polypeptides. They are synthesized through living cationic ring-opening polymerization, LCROP, which provides tight control over molecular weight, narrow polydispersity, well-defined block-copolymer architectures, and diverse α- and ω-end groups that support terminal conjugation with targeting ligands (Van Guyse et al. 2024). LCROP offers broad compatibility and extensive side-chain tunability, producing chemical diversity comparable to polypeptides while avoiding structural heterogeneity typical of other biomaterials. POx and POzi backbones contain hydrogen-bond acceptor carbonyls but lack hydrogen-bond donors, preventing secondary structure formation while enabling interactions with biomolecules (Makhayeva et al. 2026; Palchak et al. 2026; Sundaramurthy et al. 2014).

The first POx-based medical product, Ethisia, a hemostatic sealing patch for internal bleeding, received European approval in 2023 (Ethicon, Inc. 2023). In parallel, Serina Therapeutics is advancing POx–drug conjugates and POx-based lipids for LNPs using hydrophilic poly(2-ethyl-2-oxazoline), EtOx, as a PEG alternative to mitigate PEG-associated immunogenicity (Golba et al. 2025; Sanchez et al. 2024; van Zyl et al. 2024). Notably, the absence of preexisting anti-POx antibodies, in contrast with the high prevalence of anti-PEG antibodies, indicates a favorable safety profile. Beyond PEG replacement, POx and POzi provide a foundation for controlled nanostructure formation and advanced drug-delivery systems (Sundaramurthy et al. 2014).

Kabanov, Luxenhofer and Jordan significantly advanced the field by developing high-drug-loaded POx and POzi polymer micelles, PMs, for diverse therapeutic agents (Hwang et al. 2020; Kabanov et al. 2016; Lubtow et al. 2017; Luxenhofer et al. 2010). The core–shell architecture solubilizes hydrophobic drugs while providing hydrophilic stabilization in aqueous media. POx and POzi PMs outperform conventional micelles across multiple parameters, including drug loading, retention, stability, and tumor accumulation. Drug loads 10–100× higher than traditional micelles enable intravenous formulations with minimal excipient content, reducing toxicity relative to surfactants such as Cremophor EL or Pluronics (He et al. 2016). At ultrahigh loadings, greater than 50% v/v, the drug contributes to micelle stability, lowering critical micelle concentration, CMC, and enabling co-encapsulation of otherwise incompatible agents (Han et al. 2012; Lim et al. 2022).

Paclitaxel, a frontline therapy for triple-negative breast cancer, forms exceptionally stable spherical POx micelles, supporting co-formulation with chemotherapeutic and immunotherapeutic agents in ways not achievable with liposomes, exosomes, or albumin-bound nanoparticles (Lim et al. 2023; Wan et al. 2019). Micelle morphology significantly affects performance, where spherical micelles demonstrate superior tumor accumulation and therapeutic efficacy compared with wormlike structures (Lim et al. 2022).

Key physical parameters, including drug loading, micelle–serum partition constants, and CMC, govern pharmacokinetics, PK, tumor distribution, and efficacy (Hwang et al. 2020). These quantifiable equilibrium properties support PK–PD modeling, mechanistic in vitro–in vivo correlations, and rational micelle design. Scalable manufacturing methods such as lyophilization and spray drying enable stable clinical-grade production. Beyond PMs, POx and POzi platforms now include polyion complexes for nucleic acid delivery, POx-lipid conjugates for LNPs, drug nanocrystals, and oral delivery systems with enhanced bioavailability.

Taken together, POx and POzi materials illustrate how designer polymer systems can reshape nanocarrier performance, providing a foundation for Sect. 4.2, which examines vascular-confined nanoconstructs engineered to concentrate thrombolytic activity within the vascular lumen for the treatment of ischemic stroke.

### Vascular-confined nanoconstructs for controlled thrombolysis

Nanocarriers designed to operate exclusively within the vascular lumen offer a promising strategy to improve thrombolysis by retaining enzymatic activity at the clot interface while minimizing systemic toxicity and neurovascular injury.

Thrombolytic therapy for acute ischemic stroke is constrained by narrow treatment windows, hemorrhagic risk, and rapid systemic inactivation of tissue plasminogen activator, tPA (De Meyer 2026). Discoidal polymeric nanoconstructs, tPA-DPNs, address these challenges by confining tPA within the vascular compartment, preserving enzymatic stability while reducing neurovascular exposure (Spano et al. 2025). Free tPA rapidly degrades and extravasates across the compromised blood–brain barrier (BBB), which increases bleeding risk and neurological damage. By contrast, discoidal nanoconstructs function as vascular-targeted depots that concentrate thrombolytic activity at the occlusion site.

In murine microvasculature, intravenous tPA-DPN recanalizes approximately 90% of occluded venules compared with roughly 40% achieved by free tPA at equivalent doses (Spano et al. 2025). Notably, even one-tenth of the tPA-DPN dose clears approximately 70% of occlusions, an important advantage for bleeding-sensitive patients. In stroke models, free tPA increases lesion volume and BBB permeability, whereas tPA-DPN preserves neurological outcomes and improves survival, approximately 75% versus 10%, reflecting enhanced neuroprotection through luminal confinement.

The discoidal geometry and deformability of the nanoconstructs enhance vascular adhesion and clot penetration relative to spherical nanoparticles (Godin et al. 2010; Moore et al. 2022; Spano et al. 2025). Shape- and mechanics-driven margination improve interaction with clot interfaces, demonstrating that nanocarrier physical properties directly influence therapeutic outcome (Godin et al. 2010; Palange et al. 2017). These vascular-confined constructs also create opportunities for co-delivery of neuroprotective agents or anti-inflammatory payloads, enabling multimodal stroke interventions with reduced systemic exposure.

Translational development will require scalable conjugation chemistry, hemocompatibility evaluation, and potential integration with catheter-based delivery workflows in thrombectomy settings. Nonetheless, vascular-confined nanomedicine underscores a broader shift in the field, where geometry, mechanics, and spatial localization function as programmable design variables rather than passive material attributes.

This emphasis on carrier geometry and vascular dynamics leads into Sect. 4.3, which explores how polymer–drug conjugates and rationally engineered material systems further expand the design space for precision nanotherapeutic delivery.

### Engineering polymer prodrugs and stimuli-responsive systems

Polymer prodrugs and stimuli-responsive nanomaterials leverage precise chemical design to improve therapeutic index, drug stability, and spatiotemporal control, forming a robust class of translational platforms for oncology, infectious disease, and long-acting drug delivery.

Stimuli-responsive polymer prodrugs have a long and foundational history in drug delivery (Duncan and Kopeček 1984; Ringsdorf 1975). Their underlying rationale centers on improving therapeutic index by coupling controlled polymer synthesis with modular drug-linker design. Advances in controlled polymerization and functional monomer synthesis now enable the integration of targeting motifs, programmable linkers, and well-defined release kinetics into unified polymer architectures (Chavas et al. 2021; López et al. 2022; Nguyen et al. 2024; Song et al. 2025). Copolymer composition, sequence control, and architectural motifs collectively dictate tissue distribution, cellular uptake, and intracellular activation pathways. Because these materials are compositionally defined and fully synthetic, they provide reproducible scaling, predictable analytical characterization, and favorable chemistry, manufacturing, and controls, CMC, profiles for clinical translation (Pottenger et al. 2024).

A recent innovation demonstrates the potential of fully synthetic polymer prodrug depots for long-acting drug delivery (Ho et al. 2021). These injectable systems rely on polymer–drug conjugation rather than matrix erosion. Upon injection, the polymer prodrug forms an in situ gel without crosslinking, allowing drug release governed by water ingress and linker cleavage kinetics. This design yields near zero-order release with minimal burst and limited pharmacokinetic tailing. Studies in rodent and non-human primate models show programmable depot durations extending to year-long release at clinically relevant doses for HIV prophylaxis (Ho et al. 2021). Release duration and daily dosing profiles are tunable through linker design, spacer length, molecular weight, and copolymer composition.

Polymer prodrugs also advance targeted immunotherapy. Mannosylated polySTING conjugates deliver engineered STING agonists to CD206-positive dendritic cells and macrophages, where enzymatic linker cleavage releases active drug intracellularly, promoting dendritic cell maturation, CD8 T-cell priming, and potent antitumor responses (Nguyen et al. 2024; Song et al. 2025). Related systems deliver antibiotics within alveolar macrophages to treat lethal pulmonary infections (Chavas et al. 2021; Su et al. 2018). Liver-directed tafenoquine polymer prodrugs similarly optimize delivery to hepatocytic pathogen reservoirs, improving therapeutic index for malaria radical cure (Pottenger et al. 2024).

Collectively, these polymer prodrug systems illustrate how precise chemical programming enables durable, tissue-specific therapies, setting the stage for Sect. 5.1, which examines advanced organ-specific genetic nanotherapies.

## Gene & RNA nanotherapies for organ-specific delivery

### In vivo CRISPR gene editing for hematologic disease

CRISPR/Cas9, introduced in 2012, has rapidly advanced biomedical research and led to major clinical milestones, including the first clinical trial in 2016 and the 2023 FDA approval of Casgevy for ex vivo gene editing therapy of sickle cell disease and beta-thalassemia (Chakraborty et al. 2026; Park and Bao 2021; Sharma et al. 2021). While this therapy highlights the success of ex vivo editing, in vivo CRISPR delivery offers broader potential by enabling direct gene editing within the body, improving access to difficult tissues, reducing costs, and positioning nanoparticle-mediated delivery systems as key to expanding therapeutic applications (Ramirez-Phillips and Liu 2021; Wei et al. 2020). Lipid nanoparticles (LNPs) provide a scalable and clinically viable platform for systemic CRISPR delivery, making them central to next-generation gene-editing strategies for hematologic disease (Park et al. 2021).

Comparative analyses of Cas nucleases form a core component of current translational efforts. Bao and colleagues systematically evaluate Cas orthologs at β-globin, BCL11A, and γ-globin loci to assess editing efficiency, specificity, and mutational signatures (Shi et al. 2023). Advanced sequencing technologies reveal complex repair outcomes, including large on-target deletions and chromosomal rearrangements, which remain undercharacterized yet clinically relevant (Yoo et al. 2026). This work highlights the need to integrate high-resolution genomic analytics into preclinical development pipelines to ensure therapeutic safety and predictability.

A parallel thrust focuses on LNP-mediated in vivo editing of HSCs. By bypassing the high cost and complexity of autologous transplantation, approximately 2.2 million US dollars per patient, in vivo gene editing aims to democratize access to genetic cures (Park et al. 2022). However, effective in vivo editing requires optimization of nanoparticle chemistry, endosomal escape mechanisms, and bone marrow tropism. Emerging approaches include HSC niche-targeting ligands, transient immune modulation to enhance engraftment, and strategies that promote homology-directed repair for precise correction (Tong et al. 2019).

These efforts establish foundational knowledge for developing clinically viable, globally accessible CRISPR-based therapies. Continued progress in nanoparticle targeting, nuclease engineering, and genomic safety profiling will accelerate the transition toward efficient, safe and affordable in vivo gene-editing treatments for hematologic disease.

These in vivo gene-editing advances naturally connect to Sect. 5.2, which examines nanoparticle strategies engineered for targeted RNA delivery to the kidney and other extrahepatic tissues.

### Kidney-targeted nanoparticles for RNA delivery

Nanoparticles engineered for selective renal accumulation enable the targeted delivery of siRNA, mRNA, and gene regulators to kidney tissues, addressing diseases historically inaccessible to systemic RNA therapeutics.

Kidney disease and related cardiovascular disorders contribute to more than ten million deaths annually (Boateng and Ampofo 2023; Murphy et al. 2016; Ndumele et al. 2023). Current therapeutic options slow disease progression but rarely reverse pathology. Consequently, technologies that enable targeted renal delivery of biologics and nucleic acids have become a major priority in nanomedicine (Lv and Zhang 2019; Trac et al. 2023; Vasylaki et al. 2024). Despite recent progress, no regulatory agency has yet approved a kidney-targeted nanoparticle therapy (Schoales et al. 2025).

Typical nanoparticles accumulate in the liver due to their sub–200 nm diameters and susceptibility to opsonization (Chen et al. 2025; Cheng et al. 2020; Huang et al. 2022). By contrast, ultra-small particles below 10 nanometers are rapidly filtered and excreted, while microparticles preferentially sequester within pulmonary capillary beds (Short et al. 1996). These constraints motivate design strategies that tune size, charge, and surface composition to improve targeting of renal tubules and the glomerulus (Cheng et al. 2020; Vasylaki et al. 2024). Nevertheless, most nanoparticle systems still exhibit dominant hepatic accumulation, underscoring the need for alternative approaches in renal drug delivery (Williams et al. 2015).

Approximately a decade ago, Williams and collaborators identified polymeric mesoscale nanoparticles, MNPs, as a kidney-selective platform (Williams et al. 2015, 2018). These 300 to 500 nm constructs preferentially accumulate in proximal tubular epithelial cells with minimal off-target uptake. MNPs have delivered siRNAs, mRNAs, peptides, small molecules, and other cargo with therapeutic efficacy demonstrated in models of cisplatin-induced acute kidney injury, chronic kidney disease, ischemia–reperfusion injury, and autosomal dominant polycystic kidney disease (Schoales et al. 2025; Vasylaki et al. 2024). Additional studies reveal benefits in diabetic kidney disease and salt-sensitive hypertension after delivery of anti-inflammatory siRNAs (Benson et al. 2022; Veiras et al. 2022).

Other nanoparticle systems seeking kidney specificity use complementary design rules, including active ligands or selective organ targeting chemistries (Schoales et al. 2025; Vasylaki et al. 2024). Future work aims to extend these platforms toward large-animal validation, scalable manufacturing, broader cargo compatibility, and expanded indications in renal inflammation, fibrosis, and metabolic disease.

These kidney-targeted RNA delivery strategies align with broader efforts in Sect. 5.3 to engineer nanocarriers for cardiac and vascular gene modulation, where tissue-specific targeting similarly governs therapeutic success.

### Cardiovascular RNA nanotherapies

Cardiovascular RNA therapeutics increasingly rely on engineered nanocarriers to overcome the unique anatomical and physiological barriers of the heart, where rapid perfusion, dense tissue architecture, and immune surveillance restrict effective delivery.

Cardiovascular diseases, including myocardial infarction, heart failure, and arrhythmias, remain the leading contributors to global morbidity and mortality (Tsao et al. 2023). Existing therapies slow progression but rarely reverse underlying damage, leaving substantial residual risk (Cooke and Youker 2022). RNA-based therapeutics hold promise for modulating the molecular pathways governing post-injury repair, cardiomyocyte protection, and pathological remodeling (Huang et al. 2022). However, effective delivery to the heart is limited by rapid blood flow, compact myocardial structure, opsonization by circulating immune cells, and restricted direct access (Sahoo et al. 2021). These barriers likely contributed to the limited clinical efficacy of AZD8601, an intracardiac VEGF-A mRNA therapy that achieved safety endpoints but not primary clinical outcomes (Anttila et al. 2023).

Lipid nanoparticles, LNPs, offer a scalable and adaptable platform to address these constraints. First, varying helper lipid chemistry tunes biophysical properties that promote passive cardiac tropism (Radmand et al. 2024). Second, engineering the protein corona on LNP surfaces can enhance myocardial accumulation by enriching or depleting specific adsorbed proteins such as ApoE (Schrader Echeverri et al. 2025; Shuvaev et al. 2025). Third, ligand functionalization with antibody fragments or peptides enables cell type–specific targeting of cardiomyocytes, fibroblasts, or immune cells central to disease progression (Paunovska et al. 2022). Fourth, image-guided transcatheter approaches can deliver LNPs directly to ischemic myocardial regions, improving local concentration while limiting systemic exposure (Handa et al. 2025). Fifth, LNPs also exert therapeutic benefit without myocardial accumulation by reducing circulating pathogenic proteins through liver-directed delivery or by generating antifibrotic immunotherapies in peripheral immune compartments (Musunuru 2022; Rurik et al. 2022).

Across these five strategies, a therapeutic framework emerges in which rational design of LNP chemistry, targeting ligands, protein corona composition, and delivery route enables precise and safe RNA modulation of cardiovascular disease.

These cardiac RNA delivery platforms connect directly to Sect. 5.4, which examines selective hepatocyte expansion strategies for durable liver genome editing.

### Repair drive liver genome editing

Selective expansion approaches leverage regenerative biology to improve the efficiency of liver gene editing, enabling durable correction of metabolic and genetic disorders.

Precise gene correction in hepatocytes is constrained by the limited frequency of homology-directed repair, HDR, which occurs in approximately 1% of cells undergoing division at a given time. To address this barrier, researchers have proposed strategies that capitalize on the regenerative capacity of the liver to enrich for corrected hepatocytes. Selective expansion systems increase the proportion of edited cells by granting them a survival or proliferative advantage, enabling repopulation of the liver with corrected genotypes (Pankowicz et al. 2018; Vonada and Grompe 2024).

Early in vivo selection methods used genetic or chemical inhibition of fumarylacetoacetate hydrolase, Fah, combined with knockdown of Hpd to eliminate uncorrected hepatocytes (Nygaard et al. 2016; Vonada et al. 2024). More recent approaches employ acetaminophen conditioning, where transgene-corrected hepatocytes survive and proliferate due to reduced or absent cytochrome P450 reductase activity (De Giorgi et al. 2025a, b; Vonada et al. 2021). These conditioning methods effectively enrich for genetically modified hepatocytes while maintaining overall liver architecture.

De Giorgi and colleagues developed an in vivo selection platform termed Repair Drive, which uses small interfering RNA (siRNA) to temporarily inhibit an essential gene, eliminating unedited and incorrectly edited cells. As proof-of-concept, adeno-associated viral delivery of CRISPR/Cas9 and donor templates targeted the Apoa1 safe harbor locus. Hepatocytes incorporating the therapeutic transgene and the recoded essential gene FAH gain a survival advantage during siRNA-mediated selection, enabling rapid expansion of corrected cells (De Giorgi et al. 2025b). Repair Drive increases HDR-corrected hepatocyte frequencies from less than 1% to approximately 25%, while restoring normal liver metabolism and physiology following siRNA washout.

These selective expansion strategies will help shift liver genome editing from simple gene disruption towards precise repair, enabling correction of a broader range of hepatic genetic disorders.

This progression toward precision editing complements Sect. 5.5, which explores additional RNA and gene-modulation approaches for extrahepatic tissues where selective expansion is not feasible.

### Transient telomerase mRNA therapy for radiation-induced skin injury

Nanoparticle-enabled mRNA therapeutics provide localized protection of normal tissues during cancer treatment, offering transient, nongenomic interventions that modulate acute injury responses without long-term integration risks.

Radiation dermatitis is a pervasive dose-limiting toxicity of radiotherapy, affecting most patients undergoing cancer treatment and lacking an approved prophylactic therapy (Huber et al. 2011). Tissue injury arises not only from nuclear DNA double-strand breaks but also from mitochondrial dysfunction, oxidative stress, and persistent senescence signaling in skin cells (Burke et al. 2022). Transient activation of telomerase reverse transcriptase, TERT, delivered via mRNA, provides a strategy to preserve cutaneous integrity (Chang et al. 2024; Li et al. 2026).

Lipid nanoparticle, LNP, mediated delivery of TERT mRNA enhances DNA repair capacity and reduces mitochondrial stress across primary keratinocytes, fibroblasts, endothelial cells, and ex vivo human skin (Li et al. 2026). Treatment suppresses radiation-induced γH2A.X accumulation, apoptosis, mitochondrial reactive oxygen species, and membrane potential loss, while decreasing base damage and senescence markers (Li et al. 2026). These protective effects of a single administration of TERT mRNA occurred without detectable increases in telomere length, supporting a noncanonical mechanism rooted in genome and mitochondrial maintenance rather than telomere extension (Li et al. 2026).

Delivery strategy plays a central translational role. Comparative evaluations indicate that while cationic LNPs perform efficiently in vitro, ionizable lipid-based LNPs combined with microneedling enable effective and spatially confined TERT expression in human skin explants (Chang et al. 2024). This pairing aligns with existing dermatologic and oncologic workflows and highlights the broader shift toward brief, tissue-targeted mRNA therapeutics capable of modulating acute injury responses while limiting off-target exposure.

Looking forward, transient TERT mRNA therapy represents a versatile platform for mitigating normal tissue toxicity during radiotherapy (Mojiri et al. 2021; Qin et al. 2025). Beyond radiation dermatitis, similar strategies may address acute genomic and mitochondrial stress in barrier tissues, expanding the role of localized, temporally controlled mRNA interventions in clinical oncology.

These insights into transient mRNA therapeutics provide a foundation for Sect. 6.1, which examines nanocarrier-based strategies designed to modulate microglial activation and deliver neuroprotective agents to inflamed neurovascular units.

## Nanomedicine for neurological disorders & BBB interfaces

### Liposomal dexamethasone & siRNA nanoparticles for neuroinflammation

Nanocarrier-based strategies that modulate microglial activation, deliver neuroprotective agents, or reprogram dysfunctional neuroimmune networks offer a promising avenue for treating neurodegenerative diseases.

Microglial dysfunction is increasingly recognized as a mechanistically actionable axis in Alzheimer’s disease (AD) beyond classical amyloid and tau pathology (Lull and Block 2010). In aging and AD, microglia gradually lose homeostatic surveillance and phagocytic capacity while adopting proinflammatory programs that amplify oxidative stress and cytokine signaling. These maladaptive states accelerate synaptic injury and neuronal loss. Reducing microglial overactivation is therapeutically compelling yet highly constrained by delivery challenges, since less than 1% of a systemically administered dose typically reaches the brain parenchyma (Howard et al. 2017).

Nanocarriers provide a rational strategy to overcome these exposure limitations while enabling cell-state-selective immune modulation. By stabilizing labile payloads, tuning pharmacokinetics, and enabling spatiotemporal control of release, nanoplatforms concentrate therapeutics within inflamed neurovascular units while reducing off-target immunosuppression (Battaglini et al. 2024; Cerqueira et al. 2020). Design parameters, including carrier size, surface chemistry, and degradation kinetics, govern the ability to access or exploit blood-brain barrier (BBB) transport pathways (Roney et al. 2010). BBB endothelial and perivascular compartments express receptor systems suitable for receptor-mediated transcytosis, enabling selective enrichment at sites of neuroinflammation (Li et al. 2021). In this context, nanocarriers function not merely to achieve brain entry but also to modulate specific injury niches, delivering anti-inflammatory molecules, redox-active antioxidants, or transient genetic regulators that reset maladaptive microglial states (Zhao et al. 2020).

We previously demonstrated that biomimetic leukocyte-derived nanoparticles accumulate within inflamed brain tissue after systemic administration, reducing macrophage infiltration and lesion burden following traumatic brain injury (Zinger et al. 2021). In addition, liposomal dexamethasone suppresses reactive gliosis in a sex-dependent manner (Baudo et al. 2024), while systemically delivered lipid nanoparticles (LNPs) carrying neuroprotective mRNA further attenuate inflammatory signaling and oxidative stress after traumatic brain injury (Kara et al. 2026). Given the epidemiologic association between neuroinflammation and dementia risk (Boulton and Al-Rubaie 2025), microglia-centered nanotherapies are increasingly positioned as plausible disease-modifying interventions in AD and related disorders (Kara and Villapol 2026; López-Espinosa et al. 2024).

These neuro-targeted strategies lead naturally to Sect. 6.2, which examines the use of structurally robust polymersomes to transport complex biologics across the BBB.

### Polymersomes for delivery of biologics across neurologic barriers

Polymersomes have emerged as structurally robust vesicular nanocarriers capable of transporting biologic therapeutics across restrictive neurologic barriers, leveraging their tunable membranes, controlled degradation profiles, and modular surface chemistry.

Polymersomes are increasingly used for biologic drug delivery because they incorporate block-copolymer membranes with high structural stability and versatile cargo-loading capacity (Leong et al. 2018). Unlike conventional polymeric nanoparticles, polymersomes possess a bilayer analogous to liposomes but formed through hydrophilic blocks on the inner and outer surfaces and a hydrophobic membrane core. The increased membrane stability arises from hydrophobic polymer chain entanglement (Discher and Ahmed 2006), which enables simultaneous encapsulation of hydrophilic cargo in the lumen and hydrophobic agents within the membrane (Foster et al. 2025). This structural stability supports the delivery of proteins, nucleic acids, and other high–molecular-weight biological therapeutics (Iqbal et al. 2020).

Release profiles of polymersomes are programmable through rational polymer chemistry. Degradation kinetics can be tuned through polymer backbone selection, allowing responsiveness to acidic conditions (Kelly et al. 2017), enzyme-rich environments (Foster et al. 2024; Paruchuri et al. 2022), thermal shifts (Surapaneni et al. 2023), or external stimuli such as light (Shao et al. 2020). These features support targeted therapeutic release within specific physiologic compartments.

Crossing the blood–brain barrier, BBB, and blood–nerve barrier, BNB, remains a significant challenge for biologic therapies. Surface functionalization of polymersomes with proteins or peptides has demonstrated success in enhancing translocation across these barriers. Apolipoprotein E–based ligands target inflammatory receptor upregulation in neurologic injury models (Han et al. 2025). Polymersomes conjugated with rabies virus glycoprotein enhance retrograde transport, supporting delivery across both the BBB and BNB (Huey et al. 2017; Trumbull et al. 2025). Transferrin receptor–targeted polymersomes similarly improve brain uptake through receptor-mediated transcytosis (Gao et al. 2010). In intranasal applications, untagged PEG coatings can improve mucopenetration and enhance delivery to the brain (Xu et al. 2015).

Polymersomes demonstrate significant potential as vehicles for targeted biologic delivery across neurologic barriers. Future platforms will likely integrate disease-specific targeting ligands, responsive linkers, and therapeutic payload combinations to address pathologies such as neurodegeneration, nerve injury, and CNS infection.

These polymersome-based approaches for CNS delivery provide a conceptual link to Sect. 7.1, which introduces bio-integrated vascular devices that combine structural support with diagnostic nanoparticle functionality.

## Nanotheranostics and imaging-driven delivery

### Bioresorbable vascular devices with nanoparticle theranostics

Bioresorbable vascular implants enhanced with diagnostic nanomaterials represent an emerging class of theranostic devices that integrate mechanical support, localized drug delivery, and real-time imaging to monitor vascular remodeling and device degradation.

Metallic vascular implants provide essential structural support for endovascular procedures, yet they remain limited by thrombosis, chronic inflammation, device migration, and the need for retrieval once the period of clinical risk has passed (Huang et al. 2017). Bioresorbable polymers address several of these limitations by offering temporary mechanical stabilization followed by controlled degradation, but their radiolucency restricts real-time monitoring. Embedding radiopaque nanoparticles into resorbable polymer matrices improves visualization under fluoroscopy and CT imaging while preserving mechanical and biochemical performance (Damasco et al. 2022; Tian et al. 2018).

One application of this strategy is the development of fully absorbable inferior vena cava filters. Conventional metallic filters require retrieval, whereas PPDO-based filters degrade naturally following risk resolution (Huang et al. 2017). Incorporating gold, bismuth, gadolinium, or tungsten nanoparticles through wet-dip coating enhances radiopacity and supports accurate placement and longitudinal monitoring without compromising clot-trapping efficiency (Damasco et al. 2022; San Valentin et al. 2024, 2025; Tian et al. 2018).

Resorbable nanocomposite systems also advance the design of perivascular wraps intended to prevent arteriovenous fistula failure. Electrospun polymeric wraps loaded with anti-inflammatory agents or radiopaque nanoparticles improve mechanical stabilization, reduce neointimal hyperplasia, and enable noninvasive imaging of vascular remodeling during AVF maturation (Barcena et al. 2023a, b, 2024, 2025b; Klusman et al. 2023). These wraps promote endothelial repair while limiting stenotic progression, demonstrating the therapeutic value of localized drug delivery combined with imaging capability.

Small-diameter vascular grafts similarly benefit from nanocomposite strategies. Diagnostic nanoparticle incorporation improves graft visibility, while embedded anti-stenotic agents and controlled degradation support endothelialization and natural vessel regeneration (Barcena et al. 2025a; Melancon et al. 2025). These systems demonstrate how structural design, degradation kinetics, and imaging compatibility function as coordinated therapeutic levers within advanced vascular implants.

Across these examples, biointegrated vascular devices unite structural function with spatiotemporally controlled imaging and therapy, enabling more personalized, adaptive, and safe endovascular interventions.

These vascular nanocomposite systems introduce broader classes of implantable and biointegrated technologies discussed in Sect. 7.2, which examines mass-spectrometry-guided spatial mapping of drug distribution in dermal nanodelivery systems.

### Mass-spectrometry-guided dermal nanodelivery systems

Spatially resolved analytical tools are becoming central to the development of dermal and transdermal nanodelivery systems, by precisely mapping drug localization within specific skin strata, ensuring therapeutic accumulation at the site of pathology.

Dermal and transdermal drug delivery rely heavily on the depth at which a therapeutic accumulates. Topical treatments require retention within the viable epidermis, whereas systemic transdermal approaches depend on permeation into the deeper dermis where vascular density increases (Parsi et al. 2011). Fungal pathogens colonize distinct skin layers, making depth-controlled delivery essential for antifungal therapy (Walsh and Dixon 1996). However, current in vitro permeation testing provides limited and indirect information on depth-dependent drug disposition (FDA 2022). Fluorescence-based visualization techniques offer partial solutions but often require labeled compounds or model drugs that may not reflect native pharmacologic behavior (Alvarez-Román et al. 2004; Touitou and Natsheh 2021).

Desorption Electrospray Ionization Mass Spectrometry Imaging, DESI-MSI, provides a rapid, label-free method for directly visualizing drug localization within skin layers (D’Alvise et al. 2014; Eberlin et al. 2014; Feider et al. 2019; Takáts et al. 2004, 2005). A newly developed automated spatial clustering tool segments MSI data into thin, ordered skin strata beginning at the stratum corneum. This algorithm eliminates manual overlay steps, reduces analysis time to under ten minutes per specimen, and improves reproducibility while preserving spatial accuracy.

To demonstrate the utility of DESI-MSI, nanoscale delivery systems designed to target different skin depths, including ethosomes, transethosomes, and microemulsions, were evaluated for permeation of the antifungal terbinafine in human and porcine skin (Ascenso et al. 2015; Barot et al. 2012). Automated MSI analysis provided depth-resolved concentration mapping, enabling comparisons of permeation kinetics and intradermal drug distribution.

In sum, label-free DESI-MSI represents a significant advancement in the development and optimization of nanodelivery platforms for skin disease. Direct, label-free depth mapping enables rational formulation design, supports mechanism-driven carrier engineering, and minimizes systemic exposure by ensuring therapeutic localization within desired skin layers.

These analytical innovations naturally lead into Sect. 7.3, which examines molecular MRI contrast agents engineered for targeted imaging of neurodegenerative biomarkers.

### Molecular magnetic resonance imaging of neurodegenerative biomarkers

Advances in molecular magnetic resonance imaging, mMRI, enable the visualization of neurodegenerative disease biomarkers with high anatomical fidelity, supporting earlier diagnosis and a deeper mechanistic understanding of Alzheimer’s and Parkinson’s pathology.

Neurodegenerative disorders such as Alzheimer’s disease and Parkinson’s disease are characterized by the accumulation of protein aggregates including amyloid beta, hyperphosphorylated tau, and alpha synuclein, together with widespread neuroinflammation (Kelser et al. 2024). Clinical imaging of these biomarkers relies predominantly on positron emission tomography, which offers molecular specificity but is limited by cost, radiation exposure, and restricted accessibility (Young et al. 2020). Molecular MRI provides a compelling alternative by combining biomarker-targeted imaging with the high spatial resolution of MRI, provided that nanocarriers can deliver sufficiently strong contrast payloads across central nervous system barriers.

Gd(III)-based nanoparticle systems enhance mMRI sensitivity by packaging large quantities of MR-responsive nuclei into each targeting event (Tanifum et al. 2012, 2016). Engineering particle surfaces to tune water exchange and optimize relaxivity further amplifies detectable signal and enables multi-order-of-magnitude sensitivity gains (Badachhape et al. 2020). For clinical translation, these agents must demonstrate stable delivery across the blood brain barrier, predictable pharmacokinetics, and acceptable toxicological profiles.

Targeted liposomal nanoparticles encapsulating Gd(III) payloads achieve per particle relaxivity far exceeding that of conventional chelates and leverage disease-associated vascular compromise for passive access to brain parenchyma (Tanifum et al. 2014). When functionalized with amyloid beta ligands, these particles selectively label neuritic plaques and cerebral amyloid angiopathy (Badachhape et al. 2020; Tanifum et al. 2012). Alpha synuclein binding variants identify oligomeric species in cerebrospinal fluid and promote microglial uptake (Sun et al. 2024). Additional targeting strategies directed at myeloid cell surface receptors enable visualization of IBA-1 reactive cells within inflamed tissue (Sun et al. 2025b; Tanifum et al. 2020).

The nanoscale engineering strategies that enable sensitive, targeted imaging of pathological biomarkers in molecular MRI also provide a conceptual and technological foundation for interacting with biological molecules at high specificity. Building on this capability, Sect. 7.4 extends the role of nanomaterials from passive detection to active modulation of enzymatic processes, enabling programmable control of biocatalysis for therapeutic applications.

### Nanotechnology for therapeutic biocatalysis

Enzymes catalyze the chemical reactions essential for life (Bornscheuer et al. 2012; Maghraby et al. 2023). Therefore, dysregulation of enzymes is often an early hallmark of disease (Dudani et al. 2018). Accordingly, the ability to sensitively detect enzymatic activity enables early diagnosis, while precise control over enzyme function offers powerful therapeutic opportunities (Godhulayyagari et al. 2025; Pandit et al. 2024; Snider et al. 2025).

Nanotechnology provides a versatile platform to achieve both objectives by enabling programmable monitoring and regulation of enzyme-mediated biocatalysis at the molecular scale (Bhattacharya et al. 2025; Pandit et al. 2025; Sun et al. 2025a). By precisely tuning size, shape, and surface chemistry, engineered nanomaterials can interface with enzymes to modulate their structure, spatial organization, and catalytic activity within complex biological environments (Bhattacharya et al. 2025). The integration of nature’s nanomaterials, including DNA, peptides, and proteins, with synthetic nanomaterials gives rise to hybrid nano-bio platforms capable of both sensing and regulating enzymatic activity (Godhulayyagari et al. 2025; Nixon et al. 2023; Pandit et al. 2024; Snider et al. 2025).

Research in this area focuses on developing multifunctional nanostructures with three complementary capabilities. First, nanostructures are engineered to detect proteases – enzymes frequently dysregulated in cancer, neurodegeneration, and infectious disease – to enable minimally invasive diagnostic platforms by converting enzymatic cleavage events into amplified optical signals (Godhulayyagari et al. 2025; Pandit et al. 2024; Snider et al. 2025). Second, nanostructures are designed and created to bind disease-relevant enzymes and provide programmable on/off control in response to user-defined chemical cues, establishing a foundation for enzyme therapeutics that activate only under specific physiological or environmental stimuli (Godhulayyagari et al. 2025). Third, nanomaterials are tailored to enhance enzyme activity by more than 20-fold without requiring genetic or chemical modification using nanoscale confinement and microenvironment engineering to increase catalytic efficiency (Bhattacharya et al. 2025; Pandit et al. 2025; Sun et al. 2025a). When combined with enzymes, these materials generate therapeutic systems that outperform native enzymes in killing multidrug-resistant bacteria and inhibiting biofilm formation (Sun et al. 2025a).

Collectively, these advances establish a unified framework for therapeutic biocatalysis driven by designer nanomaterials, opening new avenues for precision diagnostics, adaptive therapies, and personalized medicine.

These advances in nanotechnology-enabled control of enzymatic activity provide a foundation for designing responsive therapeutic systems that can operate dynamically within complex biological and physiological environments. This concept naturally extends to Sect. 8 in which strategies based on extracellular vesicles, cell organelles (e.g. mitochondria) and cell-derived therapies are described.

## Extracellular vesicles, organelles & cell-derived therapies

### Mechanotransduction-enhanced nanotherapeutics for targeting circulating tumor cells

Mechanotransduction within the circulatory and lymphatic systems offers a unique opportunity to enhance the efficacy of nanotherapeutics designed to eliminate circulating tumor cells (CTCs) during metastasis formation.

Circulating tumor cells experience fluid shear stress, FSS, as they transit through the bloodstream. Physiological levels of FSS significantly sensitize many cancer cell types to apoptosis induced by TNF-related apoptosis-inducing ligand, TRAIL (Hope et al. 2019; Mitchell and King 2013). This mechanosensitization has motivated the development of nanocarrier platforms that deliver apoptosis ligands under flow, converting hemodynamic forces into therapeutic advantage.

One embodiment links TRAIL and E-selectin to the surface of nanoscale liposomes, generating constructs that mimic leukocyte adhesion and selectively bind to tumor cells under shear. These “TRAIL-coated leukocytes” reduce viable CTCs, inhibit metastasis formation in prostate and colorectal cancer, and extend survival in aggressive breast cancer models (Chandrasekaran et al. 2016; Jyotsana et al. 2019; Mitchell et al. 2014; Wayne et al. 2016). Platelet interactions provide a complementary targeting route. Tumor cells naturally associate with platelets, allowing therapeutic engagement through genetic expression of platelet-bound TRAIL or synthetic platelet-mimetic particles coated with platelet membrane and exogenous TRAIL (Li et al. 2016a, b).

Chemical sensitizers further enhance TRAIL-mediated killing. Hydrophobic agents such as aspirin, low-dose chemotherapeutics, curcumin, and piperlongumine prime tumor cells to undergo apoptosis and can be co-delivered through PLGA nanoparticles or micelle-in-liposome formulations (Li et al. 2015; Sharkey et al. 2016; Zhang et al. 2020a). These integrated systems offer combinatorial control over mechanical, biochemical, and nanocarrier-mediated cues.

More recently, efforts focus on noninvasively delivering mechanical stimuli such as focused ultrasound to synergize with TRAIL-based nanomedicines, creating localized mechanotransduction that amplifies therapeutic potency (Fabiano et al. 2025).

This mechanobiology-guided paradigm demonstrates how physical forces, receptor-ligand targeting, and nanocarrier engineering converge to eliminate CTCs with high specificity, establishing a foundation for next-generation antimetastatic interventions.

These flow-activated therapeutic systems naturally lead into Sect. 8.2, where complementary nanotechnologies address metastatic niches through immune modulation and targeted molecular delivery.

### Programmable extracellular vesicles and mimetic nanocarriers for in utero therapy

Prenatal therapeutic delivery imposes uniquely stringent biological and safety constraints, motivating the development of nanocarriers capable of regulated biodistribution, selective fetal engagement, and minimal maternal-fetal risk.

Congenital anomalies remain a major driver of perinatal morbidity and mortality, yet therapeutic options are limited by the fragility of the intrauterine environment (Corradetti et al. 2024). Effective prenatal intervention requires delivery platforms that balance potency with controlled fetal exposure (Phinney and Pittenger 2017). Extracellular vesicles, EVs, and EV-inspired nanotechnologies provide a biologically coherent solution due to their ability to transfer functional cargos while retaining molecular signatures of their parental cells (Bertozzi et al. 2023; Corradetti et al. 2021).

Amniotic fluid–derived mesenchymal stromal cells, AF-MSCs, encode pregnancy-specific immunomodulatory and developmental programs that are partially mediated through EV-associated coding and noncoding RNAs (Riazifar et al. 2017; Tong and Chamley 2015; Vaiasicca et al. 2024). The coupling between parental cell phenotype and EV cargo establishes a mechanistic rationale for AF-MSC-derived EVs as cell-free prenatal delivery systems, offering tissue compatibility and immunologic tolerance (Vaiasicca et al. 2024, 2025). Preclinical studies show that AF-MSC EVs can be administered safely throughout gestation with no detectable maternal toxicity and with biodistribution patterns that include localization to gestational tissues (Fonteles et al. 2024).

Translation of native EVs, however, is challenged by intrinsic heterogeneity and low production yield (Pisano et al. 2020). To address these barriers, EV-mimetic nanovesicles, MIMs, have been engineered to replicate essential EV features while enabling improved scalability, batch consistency, and controlled loading of defined cargos, including small molecules (Sundaram and Aryal 2025) and mRNA (Villarreal-Leal et al. 2021). MIMs exhibit biodistribution profiles similar to EVs, including uterine accumulation, yet ex vivo embryo culture studies demonstrate preferential localization to the yolk sac rather than deeper embryonic tissues. This divergence suggests that EVs and MIMs may serve complementary therapeutic roles, with EVs suited for deeper embryonic penetration and MIMs offering controlled exposure profiles.

Together, AF-MSC EVs and MIMs constitute a programmable toolkit for prenatal nanotherapy, balancing biological specificity, scalable manufacturing, and finely regulated fetal delivery.

These in utero delivery platforms provide a conceptual bridge to Sect. 8.3, which extends the therapeutic use of natural and biomimetic vesicles to postnatal regenerative and immunomodulatory applications.

### Hybrid extracellular vesicle–nanocarrier platforms for targeted cancer therapy

Extracellular vesicles (EVs) serve as biological templates for hybrid engineered delivery systems, combining intrinsic homing capabilities with synthetic robustness to improve therapeutic potency in oncology.

EVs mediate intercellular communication through membrane proteins that preserve the identity and targeting behavior of their parental cells (Sundaram and Aryal 2025). Intracellular communication maintains tissue and organ evolution across species, driven by cell-borne nanoparticulate chaperones that target cells and deliver the necessary information. These chaperones are cell-released membrane vesicles, broadly defined as extracellular vesicles (EVs), which are differentiated based on their biogenesis, release pathways, size, content, and function (Abello et al. 2019; Becker et al. 2016; Behzadi et al. 2017; Mulcahy et al. 2014; Pitchaimani et al. 2018, 2019; Rayamajhi et al. 2019, 2020, 2023; Sulthana et al. 2024; Sundaram and Aryal 2025). The content of EVs consists of lipids, nucleic acids, and proteins associated with the plasma membrane, organelles, and cytosol. Due to the presence of membrane-bound and cytosolic signature proteins, EVs are well equipped to recognize target cells and are therefore highly attractive for targeted drug delivery technologies (Becker et al. 2016).

However, clinical translation is challenging due to inherent heterogeneity, limited isolation yields, and colloidal instability (Rayamajhi et al. 2023). To address these issues, re-engineering approaches have been developed using EVs or cell membrane vesicles derived from cancer cells and immune cells such as natural killer (NK) cells (Pitchaimani et al. 2018; Rayamajhi et al. 2019, 2023; Sulthana et al. 2024). These vesicles typically range from 50 to 300 nm in size and contain surface proteins such as CD63, HSP70, TSG101, ALIX, and NKG2D, which enhance targeting and cellular interaction (Pitchaimani et al. 2018, 2019; Rayamajhi et al. 2019).

By integrating EVs with synthetic liposomes, hybrid re-engineered vesicles can be formed that exhibit unimodal size distribution and improved stability (Sulthana et al. 2024). These hybrid nanoparticles demonstrate enhanced tumor accumulation and rapid cellular uptake, as verified using near-infrared dyes and gadolinium-based imaging probes (Abello et al. 2019; Rayamajhi et al. 2020; Sulthana et al. 2024).

Overall, this biomimetic strategy combines the targeting capability of EVs with the structural advantages of synthetic nanoparticles, offering a promising approach to improve tumor-specific drug delivery while minimizing off-target effects (Sulthana et al. 2024).

This modular bio-synthetic architecture enables integration of diverse therapeutic payloads, ranging from chemotherapeutics to siRNA and immunomodulators. Hybrid EV systems also reduce premature drug leakage and improve colloidal robustness, two persistent challenges for native EV formulations. These features position hybrid EV–nanocarrier platforms as versatile vectors capable of leveraging biological signatures while meeting engineering requirements for predictable performance. In doing so, hybrid platforms illustrate how combining EV biology with synthetic nanotechnology expands the therapeutic landscape for cancer treatment through enhanced targeting, improved pharmacokinetics, and biologically compatible delivery.

These hybrid EV platforms introduce Sect. 8.4, where organelle-based carriers, particularly mitochondrial nanotherapies, extend the frontier of bioengineered subcellular delivery systems.

### Mitochondria-containing evs for ischemic stroke and neurodegenerative disease

Mitochondrial dysfunction is a convergent driver of ischemic, neurodegenerative, and neuromuscular disorders, positioning mitochondria-containing extracellular vesicles as a compelling next-generation platform for metabolic rescue.

Large extracellular vesicles (EVs), derived from brain endothelial cells, BECs, contain functional mitochondria and represent a naturally evolved vehicle for organelle transfer (Dave et al. 2023a, b). BECs are particularly suited as donor cells due to their high mitochondrial density relative to peripheral endothelial populations (Oldendorf et al. 1977). These large EVs support metabolic restoration in injured neurovascular cells by delivering respiring mitochondria that integrate with host mitochondrial networks (D’Souza et al. 2021; Dave et al. 2022, 2023a, b; Dave et al. 2024).

In vitro, large EVs increase mitochondrial respiration, reduce metabolic collapse, and improve survival of BECs following oxygen glucose deprivation (D’Souza et al. 2021; Dave et al. 2022, 2023a, b; Dave et al. 2024). Ex vivo studies demonstrate mitochondrial transfer to cortical and hippocampal neurons, indicating a broad capacity for cellular uptake across multiple neural subtypes (D’Souza et al. 2021; Dave et al. 2022, 2023a, b; Dave et al. 2024; Pinky et al. 2026). In vivo, intravenously administered large EVs delivered two hours after transient middle cerebral artery occlusion significantly reduce brain infarct volume and improve neurological function (Dave et al. 2024). Intramuscular administration also enables dose-dependent delivery of labeled mitochondria to spinal motor neurons, highlighting potential applications for degenerative neuromuscular conditions such as ALS (Pinky et al. 2026).

Large EVs therefore constitute a platform for organelle-based therapeutics that restore mitochondrial oxidative phosphorylation and support cell survival across ischemic and degenerative pathologies (Liu et al. 2022a). However, translation requires mechanistic insight into EV biogenesis pathways, mitochondrial encapsulation, and determinants of mitochondrial quality control (Manickam et al. 2026). Additional studies must define the proportion of polarized mitochondria in each EV preparation, optimize colloidal stability during storage, and map biodistribution and pharmacokinetics following systemic or local administration (Dave et al. 2023a, b). Safety profiling, including immunogenicity after repeated dosing, remains essential for clinical readiness.

Collectively, mitochondria-containing EVs represent a transformative direction in regenerative nanomedicine by enabling direct restoration of cellular energy systems rather than indirect metabolic modulation.

These organelle-based repair strategies set the foundation for Sect. 8.5, where additional bioengineered vesicle platforms extend metabolic and immunologic modulation into broader therapeutic contexts.

### Exogenous mitochondrial transfer to reprogram macrophage metabolism in cardiometabolic disease

Restoring mitochondrial function in pro-inflammatory macrophages (M1 macrophages) is a promising therapeutic strategy for cardiometabolic and inflammatory diseases, targeting aberrant bioenergetics to halt chronic tissue injury.

Mitochondrial dysfunction contributes to the progression of cancer, cardiovascular disease, neurodegeneration, and metabolic disorders (Hanahan and Weinberg 2011; Zong et al. 2024). These conditions share impaired mitochondrial quality control, reduced oxidative phosphorylation, elevated oxidative stress, and dysregulated mitochondrial fusion, fission, and mitophagy dynamics (Misrani et al. 2021; Scheffer et al. 2022). In atherosclerosis, proinflammatory M1 macrophages exhibit heightened glycolysis that sustains a chronic inflammatory milieu within vulnerable plaques (Dolfi et al. 2021). Likewise, macrophage polarization toward inflammatory phenotypes drives progression of nonalcoholic fatty liver disease and its advanced form, nonalcoholic steatohepatitis (Xu et al. 2021). These observations highlight the therapeutic importance of restoring mitochondrial competence in pathological macrophage subsets.

Exogenous mitochondrial transfer has recently shown potential to reprogram metabolically impaired cells. Blanco and colleagues demonstrated that transplantation of donor mitochondria into metabolically-compromised cells enhances oxidative phosphorylation, increases oxygen consumption rate, and decreases glycolytic flux while suppressing cellular disease processes (Baudo et al. 2023; Liu et al. 2022b; Wu et al. 2018). In murine models of atherosclerosis, intravenously delivered mitochondria localize to aortic plaques and co-distribute with macrophages, coinciding with reductions in plaque burden (Liu et al. 2022b). The therapeutic effect extends systemically, with improvements observed in hepatic steatosis, inflammation, and overall NAFLD activity score following mitochondrial delivery (Liu et al. 2022b).

In the above study, polymer functionalization of isolated mitochondria provided the opportunity for incorporation of imaging probes to better evaluate in vivo performance and therapeutic localization (Liu et al. 2022b). These engineered mitochondria were previously shown to maintain respiratory activity and demonstrated enhanced internalization in cells (Wu et al. 2018). The ability to introduce functionalizable, bioenergetically competent mitochondria directly into dysfunctional innate immune cells represents a mechanistically targeted approach to counteract metabolic disease progression (Liu et al. 2022b).

Collectively, these findings position mitochondrial transplantation as an emerging class of metabolic nanotherapy capable of reversing macrophage-driven inflammation across cardiometabolic diseases.

These mitochondrial transfer strategies conclude Sect. 8 and transition into Sect. 9, which situates subcellular and vesicle-based nanotherapies within broader translational frameworks for manufacturing, safety, and clinical implementation.

## Manufacturing and quality-by-design for next-generation nanotherapeutics

As nanotherapeutics expand in molecular complexity, robust manufacturing and purposeful quality frameworks are essential to ensure reproducibility, safety, and a scalable clinical supply.

Nanomedicine pipelines increasingly incorporate small molecules, peptides, proteins, plasmid DNA, mRNA, and oligonucleotides into diverse nanoparticle architectures (Mitchell et al. 2021). This rise in structural and functional complexity demands coordinated Chemistry, Manufacturing and Controls, CMC, strategies that unify process design with analytical development. A phase-appropriate CMC roadmap de-risks translation by defining unit operations, control strategies, and analytical requirements from early development through clinical manufacturing(ICH 2021). Typical progression includes process implementation, followed by development and scale-up, demonstration batches to support toxicology and stability studies, and eventually GMP clinical manufacturing guided by validated quality control and stability programs.

Quality-by-Design, QbD, provides a structured pathway to embed quality directly into nanotherapeutics. The process begins with establishing a Quality Target Product Profile, QTPP, identifying critical quality attributes, CQAs, and linking these to critical process parameters, CPPs, through risk assessment and experimental mapping (ICH 2008, 2009, 2023). Common CQAs include particle size distribution, composition, impurity profile, and microbiological attributes. By defining how CPPs such as mixing intensity, solvent ratios, temperature, flow conditions, and downstream purification influence CQAs, QbD frameworks reduce late-stage failure risk and limit the need for costly bridging studies.

Scalability is equally central to clinical readiness. Translating milliliter-scale research processes into multi-liter manufacturing requires selection of appropriate batch or flow modalities, each governed by unique self-assembly and mass-transfer constraints (Saad and Prud’homme 2016; van Eldijk and Bänziger 2026). Flow-based systems, for example, exert strong influence on lipid and polymer nanoparticle formation. Tangential flow filtration, TFF, has emerged as a versatile purification platform, enabling efficient removal of unencapsulated drug and process impurities while supporting diverse nanoparticle classes.

Collectively, integration of QbD principles, scalable unit operations, and advanced analytical characterization provides the foundation needed to reliably produce clinical-grade nanotherapeutics suited for increasingly complex therapeutic applications.

Together, these manufacturing and quality frameworks complete the perspective by connecting scientific innovation to the infrastructure required for successful clinical translation of nanomedicine.

## Conclusion and perspective

The technologies showcased across the NanoDDS 2025 keynote and oral presentations collectively define a new era in biomedical nanotechnology, one driven by the convergence of precision delivery, immune engineering, cellular augmentation, and quantitative analytics. Emerging platforms now achieve organ- and cell-specific targeting in the heart, kidney, liver, and brain, while localized immunotherapy depots and implantable microdevices increasingly replace the systemic toxicities that have historically constrained oncology and infectious disease therapies. Mitochondrial transfer systems, extracellular vesicles, and hybrid bio-nano constructs introduce a class of “living therapeutics” that directly modulate cellular metabolism and communication. Parallel advances in quantitative nano-flow cytometry, molecular MRI probes, and image-integrated drug carriers are establishing the analytical foundation required to guide rational design and accelerate translation.

Across these domains, a deeper transformation becomes clear: nanoparticles are engineered to behave like cells, cells function as therapeutic devices, devices operate as immunomodulators, and imaging technologies evolve into pharmacologic tools. Biomaterials, in turn, are increasingly programmable, adaptive, and responsive to biological cues. The future landscape of nano- and micro-devices will be shaped by platforms that are multifunctional, image-visible, self-modulating, and organ-specific, with manufacturability and regulatory readiness embedded from inception. Ultimately, the trajectory established at NanoDDS 2025 confirms that this transition is not theoretical but actively unfolding.

## Data Availability

No datasets were generated or analysed during the current study.
